# Assessing focus through ear-EEG: a comparative study between conventional cap EEG and mobile in- and around-the-ear EEG systems

**DOI:** 10.3389/fnins.2023.895094

**Published:** 2023-09-26

**Authors:** Gabrielle Crétot-Richert, Maarten De Vos, Stefan Debener, Martin G. Bleichner, Jérémie Voix

**Affiliations:** ^1^École de technologie supérieure (ÉTS), Université du Québec, Montréal, QC, Canada; ^2^Stadius, Department of Electrical Engineering, Faculty of Engineering Sciences & Department of Development and Regeneration, Faculty of Medicine, KU Leuven, Leuven, Belgium; ^3^Neuropsychology Lab, Department of Psychology, University of Oldenburg, Oldenburg, Germany; ^4^Research Center for Neurosensory Science, University of Oldenburg, Oldenburg, Germany; ^5^Neurophysiology of Everyday Life Group, Department of Psychology, University of Oldenburg, Oldenburg, Germany

**Keywords:** EEG, brain computer interface, cEEGrid, attention, cognitive workload, working memory, machine learning, ear-EEG

## Abstract

**Introduction:**

As our attention is becoming a commodity that an ever-increasing number of applications are competing for, investing in modern day tools and devices that can detect our mental states and protect them from outside interruptions holds great value. Mental fatigue and distractions are impacting our ability to focus and can cause workplace injuries. Electroencephalography (EEG) may reflect concentration, and if EEG equipment became wearable and inconspicuous, innovative brain-computer interfaces (BCI) could be developed to monitor mental load in daily life situations. The purpose of this study is to investigate the potential of EEG recorded inside and around the human ear to determine levels of attention and focus.

**Methods:**

In this study, mobile and wireless ear-EEG were concurrently recorded with conventional EEG (cap) systems to collect data during tasks related to focus: an N-back task to assess working memory and a mental arithmetic task to assess cognitive workload. The power spectral density (PSD) of the EEG signal was analyzed to isolate consistent differences between mental load conditions and classify epochs using step-wise linear discriminant analysis (swLDA).

**Results and discussion:**

Results revealed that spectral features differed statistically between levels of cognitive load for both tasks. Classification algorithms were tested on spectral features from twelve and two selected channels, for the cap and the ear-EEG. A two-channel ear-EEG model evaluated the performance of two dry in-ear electrodes specifically. Single-trial classification for both tasks revealed above chance-level accuracies for all subjects, with mean accuracies of: 96% (cap-EEG) and 95% (ear-EEG) for the twelve-channel models, 76% (cap-EEG) and 74% (in-ear-EEG) for the two-channel model for the N-back task; and 82% (cap-EEG) and 85% (ear-EEG) for the twelve-channel, 70% (cap-EEG) and 69% (in-ear-EEG) for the two-channel model for the arithmetic task. These results suggest that neural oscillations recorded with ear-EEG can be used to reliably differentiate between levels of cognitive workload and working memory, in particular when multi-channel recordings are available, and could, in the near future, be integrated into wearable devices.

## 1. Introduction

The push to develop and democratize useful neurotechnology tools and devices has been fueled by both the academic and industrial sectors. Over the last twenty years, neuroscience as a field of research has grown remarkably, generating major funding and institutional support (Zivkovic, 2015). The private sector has also increased investments and a handful of companies are now considered leaders of innovation in the neurotech landscape, not without growing ethical concerns and public scrutiny (Jarchum, 2019; Pfotenhauer et al., 2021; Wexler, 2021). In this context, new applications are emerging for medical as well as consumer interests and with these come new challenges, specifically, the need for improved mobility (Debener et al., 2012; Gramann et al., 2014). Transferring knowledge and technology from a laboratory environment to real-world applications is both necessary and far from trivial. Electroencephalography (EEG) is a proven technique to record brain-electrical activity with the important advantage of high temporal resolution. Non-invasive EEG uses electrodes placed on the scalp to capture electrical potentials emitted by large groups of neurons firing synchronously. EEG is a safe, low-cost and low energy technology compared to other brain imaging techniques, such as magnetic resonance imaging (MRI). This has made EEG particularly appealing for brain-computer interfaces (BCI). BCIs allow humans to control electronic devices, such as computers or prosthetics (Hochberg et al., 2006), using their thoughts. Earlier uses of such devices mainly focused on communication and control for individuals suffering from different forms of paralysis (Birbaumer et al., 1999; Wolpaw et al., 2002; Vansteensel et al., 2016). However, other, more quotidian BCI applications are emerging (Zander and Kothe, 2011). For instance, BCI applications could monitor the mental state of a person in view of workplace security (Müller et al., 2008; Aricò et al., 2016; Mijović et al., 2017) and productivity, by preventing interruptions that could be detrimental to task completion and quality (Jenkins et al., 2016). If we could reliably decode when a person is focused on a task, we could protect that state of “flow” by limiting both visual and auditory distractions. This study intends to do the former using non-invasive, discrete technology that will not limit movement.

The robustness of BCI devices is improving thanks to recent progress in signal processing, machine learning, electrode technology and open-source software tools (Delorme and Makeig, 2004; Popescu et al., 2007; Blankertz et al., 2011; Gramfort et al., 2014). However, several challenges still need to be addressed. For instance, the recording equipment usually involves caps or headbands, making it unsuitable in social settings while the electrodes are usually wire-connected to bulky amplifiers making the devices cumbersome and stationary. Thus, recent EEG technological developments have been focusing on lighter equipment with fewer electrodes and greater mobility, more adapted for exploration of cognitive processes in realistic environments and situations (Casson et al., 2010; Chi et al., 2011; Debener et al., 2015; Goverdovsky et al., 2017). Wireless EEG amplifiers are becoming available and their capability to record reliable EEG data has been scientifically proven (Debener et al., 2012; De Vos et al., 2014b; Lin et al., 2014).

Ear-EEG refers to the recording of brain-electrical activity from electrodes placed in or near the ear, as opposed to traditional scalp EEG, for which electrodes are commonly placed in concentric circles on caps which cover the entire scalp. The main appeal of ear-EEG for the proposed mental focus application is that it can be inconspicuous to wear in public settings. Also, the area around the ear and inside the ear canal is usually hairless, an advantage for electrode-skin contact with or without conductive gel. Furthermore, the irregular geometry of the ear canal would allow fitted earpieces to keep the device firmly in place, thereby potentially reducing artifacts during natural movement. The first proof-of-concept for ear-EEG was published in 2011 (Looney et al., 2011) and since then, multiple research groups have demonstrated its potential, with electrodes placed around the ear, in the concha and inside the ear canal (Debener et al., 2012; Kidmose et al., 2012; Bleichner et al., 2015). Ear-EEG has already proven its capacity to reliably record auditory attention (Bleichner et al., 2016; Mirkovic et al., 2016), event-related potential (ERP) components such as the P300 (Debener et al., 2015; Pacharra et al., 2017) and factors like fatigue and sleep quality (Looney et al., 2014; Mikkelsen et al., 2018; Sterr et al., 2018). Some studies have considered the potential of ear-EEG to record alpha frequencies (rhythmical activation of the brain oscillating between 8 and 12 Hz) (Debener et al., 2015; Mikkelsen et al., 2015), but ear-EEG research has generally focused on the analysis of time-locked events. ERPs are induced potentials that are seen in response to sensory, cognitive or motor events. They combine positive and negative amplitude peaks occurring over specific time windows after stimulus onset, some are large in amplitude and identifiable even at the single trial level (e.g., P300 or N100). Another type of brain activity, which has received perhaps less attention in ear-EEG research, is continuous EEG and its oscillatory behaviors (Buzsaki, 2011). Neural oscillations are defined as power spectral densities over specific frequency ranges, delta (1–3 Hz), theta (4–7 Hz), alpha (8–12 Hz), beta (13–30 Hz) and gamma (> 30 Hz). They have been found to reflect mental states such as memory (Klimesch et al., 1996), attention (Foxe and Snyder, 2011), engagement (Berka et al., 2007) and higher thinking and reasoning processes (Palaniappan, 2006).

This study aims to investigate the EEG power spectrum extracted from signals recorded in and around the ear and to evaluate the potential to use these signals to monitor mental states. We compared conventional cap-EEG (EEG recorded over the entire head) to new devices developed for mobile ear-EEG to assess how the signals' amplitudes and power spectrum differ from one recording system to the other. The goal is to discriminate between states of high and of low focus using classification algorithms (Müller et al., 2008).

In this study, focus is associated to two main concepts: working memory and cognitive workload as seen in (Klimesch et al., 1998; Oken et al., 2006; Fougnie, 2008). *Working memory* refers to the process of using short-term memory to make immediate and conscious decisions regarding a perceptual or linguistic task. A popular paradigm used to investigate working memory is the N-back task as presented in Brouwer et al. (2012). Different working memory loads can be studied by lengthening or shortening the duration of a sequence of numbers or letters a subject is asked to remember. Working memory load is known to inversely correlate with alpha power (Owen et al., 2005; Berka et al., 2007; Regenbogen et al., 2012). Additionally, brain oscillations from theta to gamma have been found to vary according to the working memory levels (Herrmann et al., 2004; Pesonen et al., 2007). The N-back task elicits a P300 ERP, a well-known ERP component, which varies in amplitude according to task relevance and working memory (Watter et al., 2001). *Cognitive workload* relates to the difficulty and effort required to perform a task. It has strongly been associated with changes in alpha power according to the level of difficulty of the task (Anderson and Sijercic, 1996; Keil et al., 2006; Foxe and Snyder, 2011; Magosso et al., 2019). It has been reported that other frequency bands, such as delta (Harmony et al., 1996), theta (Scheeringa et al., 2008), and gamma (Shibata et al., 1999; Landau et al., 2007) also carry information relative to cognitive workload. To assess the ear-EEG's capability to detect changes in power spectral density (PSD) relevant to cognitive workload levels, an arithmetic task was replicated from established high-density cap-EEG studies (Yu et al., 2009; Rebsamen et al., 2011).

## 2. Materials and methods

### 2.1. Subjects

Fifteen subjects (nine females, six males, mean age 27.1 years, 14 right-handed) were initially recruited for this study to participate in both tasks although some participants only took part in one of the two. Each gave written informed consent and none reported any neurological or psychiatric disorders. Recruitment and procedures for this study were performed in accordance with the Ethics Committee of Oldenburg University. Subjects were remunerated at minimum wage to take part in the study.

### 2.2. Test paradigm

Two tasks were chosen to study different characteristics and common features of concentration: an N-back task to study working memory and an arithmetic task to study cognitive workload. For both tasks, subjects were seated in front of a screen where visual stimuli, letters and numbers were displayed using the Presentation® software (Version 18.0, Neurobehavioral Systems, Inc., Berkeley, CA, www.neurobs.com), which also generated event markers. The stimuli were displayed in a black font on a light gray background. For each task, subjects were first given a tutorial round to ensure the instructions were understood. Task order was balanced across subjects.

The N-back task was conducted according to the following study's procedure (Brouwer et al., 2012). Subjects memorized a series of consonants and for each new letter that appeared, they had to decide if it was a target or a non-target letter using a two-button handheld box. Button assignment for target and non-target, left or right, was balanced across subjects. The attributes of the target letter depended on the level of difficulty of the task, of which there were three. The first level or 0-back, required no working memory effort as the subjects did not have to pay attention to the sequence of letters. They only had to identify the letter 'X' as a target and any other letter as a non-target. For the 1-back, subjects needed to remember the past letter shown. If the new stimulus was the same as the previous one, one back, then that letter was a target. And finally, for the 2-back, the most demanding level in terms of working memory, the target letters were those shown two letters before, requiring the subjects to constantly update the last two letters in their head and compare it with the new one. The letters were displayed on the screen for 500 ms with a 2000 ms inter-stimulus interval during which a fixation cross was displayed at the center of the screen.

The duration of the N-back task was about 45 min. It was divided into four sessions of six two-minute blocks each. The three levels of difficulty were repeated twice during one session. Forty-eight letters were shown during each block, 33% of which were targets. The levels were given in a pseudorandom order, different for each subject. Each level was presented once before being repeated and the same level was never given twice in a row. Between each block, the subjects were shown their success rate on the screen followed by a fixation cross for 20 seconds. Subjects initiated the next block and between sessions, the experimenters would briefly interact with the subjects before initiating the next session.

The arithmetic task consisted of sums and inequalities, a procedure adapted from Rebsamen et al. (2011). Subjects were shown an addition, asked to calculate it and keep the result in mind. A new number was then displayed which could be greater, equal to or lower than the result, each with a 33% probability. Subjects were asked to compare their answer with this number, using a handheld 3-button box. The buttons were marked with the signs “<”, “=” and “>”. There was no time limit to solve a problem. The task featured five levels of difficulty; level 1: addition of two single-digit numbers, level 2: addition of a single and a double-digit number, level 3: addition of two double-digit numbers, level 4: one double and one triple-digit number and finally, level 5: an addition between two triple-digit numbers.

The arithmetic task took 35 min to complete. It was divided into two sessions, each consisting of fifteen one-minute blocks and the five levels of difficulty were repeated three times. During a block, only additions of the same level were featured. Subjects completed as many additions as they could in the one-minute time frame. The levels of difficulty were given in a pseudorandom order, different for each subject. Each level was presented once before being repeated, and the same level was never given twice in a row. Between each block, subjects were shown their success rate on the screen. A 20-s rest period followed each block, during which a fixation cross was displayed on the screen. Between sessions, experimenters would interact with the subjects until they felt ready to initiate the next session.

### 2.3. Data acquisition

**Figure 1:**
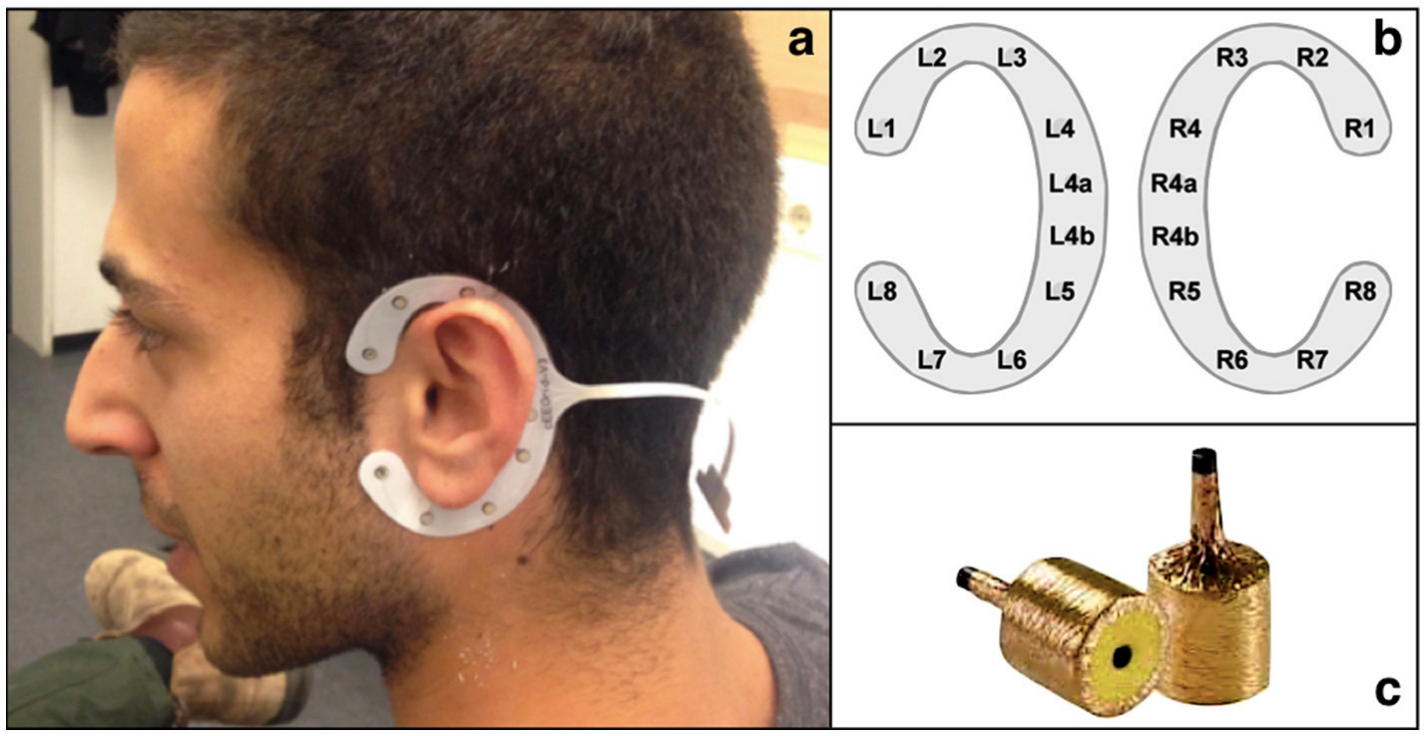
Ear-EEG recording devices: **(a, b)** show the cEEGrid; **(c)** shows the TIPtrodes^TM^.

EEG data was recorded using two recording systems concurrently: an ear-EEG mobile recording system and a high-density cap-EEG stationary recording system as described in Bleichner et al. (2016). The ear-EEG equipment consisted of a SMARTING 24-channel wireless EEG amplifier (mBrainTrain, Belgrade, Serbia) with a modified connector to two cEEGrids–concealed, around the ear self-adhesive arrays of 10 flex-printed Ag/AgCl electrodes (Debener et al., 2015) - and two TIPtrode^TM^ - in-ear foam inserts wrapped in gold foil (Bauch and Olsen, 1990) as seen in Figure 1. Ear-EEG channels, cEEGrids and TIPtrodes^TM^, were referenced to a channel on the cEEGrid placed around the subject's right ear, electrode R4b, while electrode R4a served as the analog ground. Channels from the right ear, cEEGrids and TIPtrodes (excluding R4a and R4b), were re-referenced during signal pre-processing to channel L4b ensuring that each ear-EEG channel was referenced to a channel on the contralateral ear. Data from this system was transmitted wirelessly via Bluetooth to the recording computer.

**Figure 2:**
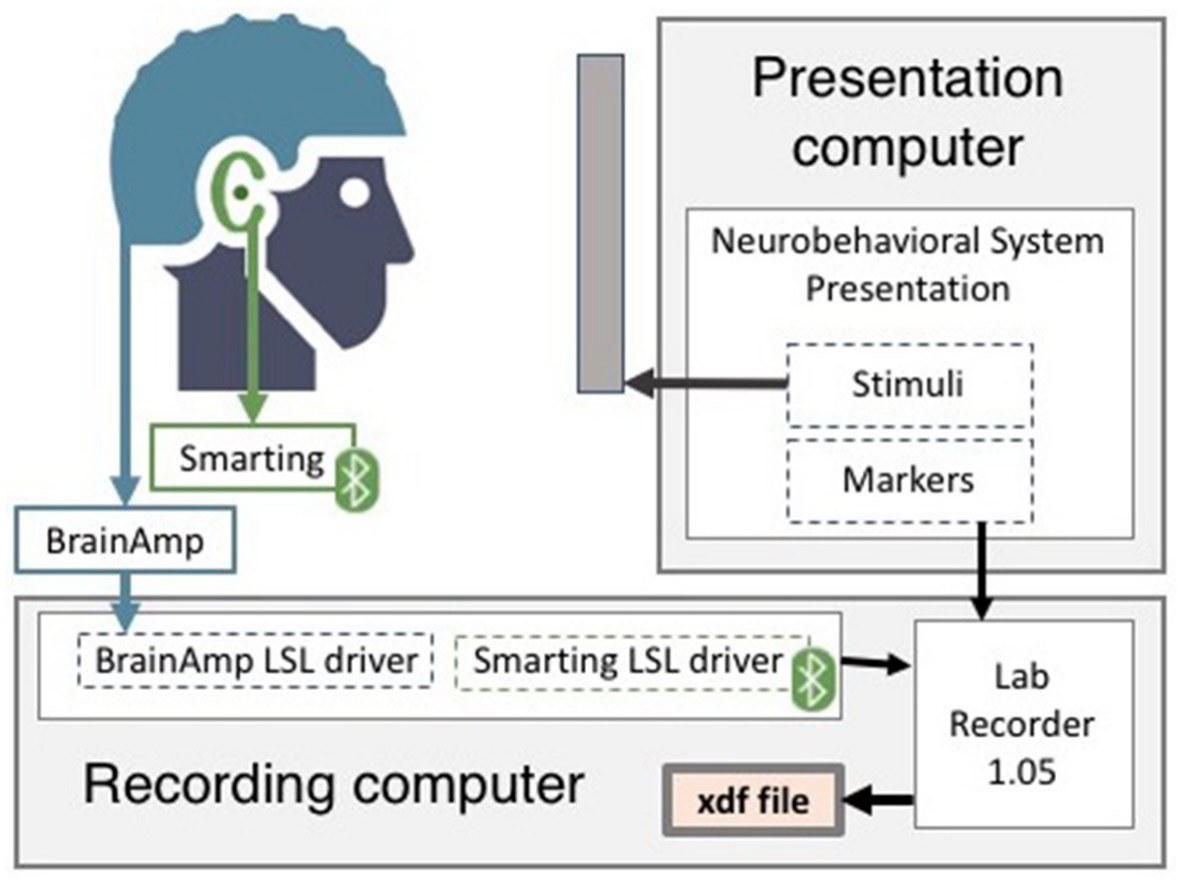
EEG devices setup and recording systems.

The cap-EEG system consisted of a research-grade EEG amplifier (Brainamp, Brainproducts GmbH, Herrsching, Germany) connected to a 96-channel Ag/AgCl EEG cap (EasyCap, Hersching, Germany) with equidistant electrodes. Twelve electrodes were not connected, that is, six over each ear, as they would have overlapped with the cEEGrids placed underneath the cap. This gave a total of 84 effective channels. The ground was placed at a central fronto-polar electrode and the reference electrode was placed at the nose-tip. This system had two dedicated eye electrodes placed under each of the participant's eyes (E29 and E30). The cap-EEG data was transmitted through fiber optic cable to the same recording computer as the one used for the ear-EEG. Both recording systems' data streams were recorded at a 500 Hz sampling rate along with a third data stream consisting of the event markers generated by the Presentation® software (Neurobehavioral Systems, Inc., Berkeley, CA, www.neurobs.com). As shown in Figure 2, they were combined into a single file, xdf format, using the Lab Recorder program from the open-source Lab Streaming Layer (LSL) data acquisition and synchronization software (Kothe, 2014).

Prior to recording, subjects were asked to wash and dry their hair. They were also given cotton swabs to clean earwax from the outer ear canal. Alcohol wipes and abrasive gel were used to prep the skin around the ear. Electrolytic gel (Abralyt HiCl, Easycap GmbH, Germany) was applied to the cEEGrid electrodes, which were then placed around the subjects' ears. Electrode impedance was adjusted to under 20 kΩ for each ear electrode. Next, the subject was fitted with the electrode head cap. The six electrode rings over each ear were taped off, all others were covered with electrolytic gel and individual electrode impedance was set to under 5 kΩ for all cap electrodes. The subjects were seated facing a screen at a distance of 1.3 meters, in a sound-proof room. Lastly, the two dry TIPtrodes^TM^ were rolled tight and inserted into the ear canal where they were given time to expand and come in close contact with the skin. Metallic clamps were attached to the gold foil of the TIPtrodes^TM^ and connected to a SMARTING adaptor along with the cEEGrids leads. The SMARTING amplifier was secured to the shoulder of the subject.

### 2.4. Data analysis

Data was analyzed offline using MATLAB (MathWorks Inc., Natick, USA) and EEGLAB, version 13.6.5b (Delorme and Makeig, 2004). Fourteen subjects took part in the N-back task and all were included in this study. Fifteen subjects were recruited for the arithmetic task, but five of these were not considered because of a stimuli marker experimental error that failed to associate EEG data blocks to their level of difficulty. EEG data was band-pass filtered between 0.5 Hz and 100 Hz and resampled at 256 Hz. The data sets were then epoched for each task. For the N-back task, epochs started 500 ms before a new stimulus was presented and ended 1500 ms after. Epochs were baseline corrected at 200 ms before stimulus presentation. This resulted in 48 epochs per block, 384 per level and 1,152 per subject. For the arithmetic task, epochs were not linked to stimuli presentation since the number of problems and the time lapse between problems varied across levels and subjects. Therefore, data from the arithmetic task was epoched at regular intervals. Regularly-spaced dummy event markers were added during pre-processing and used to epoch the EEG data in 2-second windows with a one-second overlap between windows. This produced 58 epochs per block, 348 epochs per level and 1,740 epochs per subject). Epochs from both tasks were inspected visually to select artefact-dominated trials and remove them. Less than 5% of trials were removed for each individual subject data set. An Independent Component Analysis (ICA) was then performed on all available channels for each recording equipment (84 for the cap-EEG data and 20 for the ear-EEG). The eye blink component or components were removed using topographical distribution when available and time courses of the independent component activation for both systems. Between one and three components was removed per subject per task as the eye blink component(s) for the cap-EEG data while for the ear-EEG data, components removed varied between one and two components per subject per task, with an exception of one participant for which no component were removed. This participant's ear-EEG data exhibited in fact very few blink related activity. For both the cap and the ear EEG data, the blink removal procedure was validated by inspecting superimposed single trial time courses before and after component removal using EEGLAB tools to ensure that the blinks had been removed from the signal without affecting other dynamics of the continuous EEG signal. Other components identified as noise, notably an important sinusoidal component which affected some cap-EEG electrodes located at the back of the ears and both in-ear-EEG electrodes, were removed using the ICA method as well. This noise might have been related to interference between the two recording systems and equipment.

Data sets were then processed for a spectral-domain analysis. The Power Spectral Density (PSD) was calculated for each epoch using a periodogram power spectral density estimate under a Hanning window. The results were interpreted for a frequency range from 1 to 100 Hz in 1 Hz interval bins and as band power for seven neural oscillation groups: delta (1–3 Hz), theta (4–7 Hz), alpha (8–12 Hz), a low beta band (13–19 Hz) and a high beta band (20–30 Hz), a low gamma band (30–50 Hz) and a high gamma band (50–100 Hz). This splitting of the last two frequency bands was based on the results of an analysis, not shown here, which came to a compromise between showcasing significant differences in PSD within the bands while limiting the amount of splits in the bands when the differences were not as statistically significant.

A time-domain analysis was performed for the N-back task, for which the data was resampled at 100 Hz and a low-pass filter was applied at 20 Hz to analyze the ERP response from 0 to 1,000 ms.

For both these analyses, spectral and temporal, all channels were studied and two electrodes from each recording system were selected to highlight grand average and distribution results: a centrally-located electrode, Pz for cap-EEG, L3 for ear-EEG; and another electrode which was relevant to compare in-ear EEG to cap-EEG specifically. These special electrodes were: one of the in-ear channels, E1 and E2, the two TIPtrodes^TM^ electrodes used dry inside the ear canal; and the cap-EEG channel chosen for comparison consisted of an occipital channel, O1 or O2, chosen after multiple considerations of other electrodes (midline, central and frontal pairs). The analysis (and later classification) results were found to be best at these locations which was to be anticipated considering the visual nature of both tasks' experimental stimuli. Only one in-ear channel and one occipital channel were shown in the figures since their results looked similar enough that no additional information could be gathered from representing both.

A spatial-domain analysis was performed using all 84 cap-EEG channels available. Topographical distributions for the above-mentioned seven frequency band powers was mapped for different mental load conditions. The difference between the most and least demanding conditions and the relative significance of these differences were also represented spatially.

Lastly, statistical analyses of the differences between mental load conditions was done through permutation testing (Cohen, 2014) for twelve electrodes from both recording systems which covered the recorded channels as well as possible. The cap-EEG's channels provided a good spatial distribution over the head and were based on De Vos et al. (2014b). Since the cap-EEG electrode layout used in this study differed from the cited article's layout, a correspondence was established between the 10–20 system electrode labels and the 96 equidistant electrode cap labels using the shortest Euclidean distances between electrode positions. The cap-EEG electrodes chosen for this study are: E1-Cz, E2-Fz, E5-Pz, E8-Fpz, E12-O2, E13-O1, E45-F4, E38-C4, E49-P4, E53-P3, E41-C3, E57-F3. The ear-EEG's channels which were found to be representative of the entire electrode set and overlapped with the results of prior authors (Bleichner et al., 2016). They are: L1, L2, L3, L6, L7, R1, R2, R3, R6, R7 for the cEEGrid plus the right and left in-ear TIPtrode^TM^ electrodes referred to as E1 and E2. This non-parametric test was chosen because PSD distributions from all subjects were largely non-uniform. Additionally, the statistical results of a permutation test, a z-score, is a signed indicator which gives information on the strength of the statistical test but also on the sign of the difference between conditions. It was shown that the sign of the differences - which condition yields a higher PSD or amplitude and which is lesser - changes between mental load conditions according to individual subject, frequency, time and spatial location. Each permutation test was done 5,000 times to yield z-scores for each comparison and significance threshold was placed at a p-value of 0.01, equivalent to a z-score of ± 2.326. The results of these comparisons are featured in the analysis of the EEG data for both tasks. Statistical heatmaps were created from them for both spectral and temporal domain information. To account for the important number of tests performed to produce each heatmap (over a thousand), multiple comparison correction was applied to the statistical values using the False Discovery Rate (FDR) method (Benjamini and Hochberg, 1995; Yekutieli and Benjamini, 1999).

### 2.5. Feature extraction and classification

Classification of the data epochs was performed on a subset of the total number of channels available (twelve and two) for each recording system to be consistent across both recording systems and more representative of concealed ear-EEG device constraints. Narrowing the number of channels considered was also important for dimensionality concerns associated with machine learning algorithms. Multiple models were generated using one classifier type, a step-wise linear discriminant analysis (swLDA), each model was defined by the features of the EEG signal it used. Sets of features were generated in order to compare their performance and assess the influence of factors such as the number of channels available, the PSD resolution, the cut-off frequency and the domain of the features (spectral vs temporal). The same channels as for the statistical heatmaps were used to create the twelve-channel feature sets for both recording equipment and both task. The channels used to compare in-ear to cap-EEG specifically were used to generate the two-channel features sets: E1 and E2 for the ear-EEG, O1 and O2 for the cap-EEG.

PSD features for each channel were extracted from the periodogram of the epochs generated for each task and focus level. The main PSD feature set considered was composed of PSD estimates from 1 to 100 Hz with a 1 Hz resolution also referred to as the 1 Hz frequency bin model. It generated 100 initial features per channel. Each model was trained for both cap and ear-EEG, for twelve channels (1200 initial features) and for two channels (200 initial features). The PSD values for each selected channel were concatenated.

To study the influence of PSD resolution on classifier performance, models with different PSD resolution or bin sizes were considered, two additional frequency bin models and 3 frequency band models. The highest resolution model was composed of 0.5 Hz bins, yielding 200 initial features per channels. A 5 Hz or less PSD model was evaluated, it was composed of a delta (1-3 Hz), theta (4-7 Hz) and alpha (8-12 Hz) neural oscillation bands since their frequency ranges were equal to or lower than 5 Hz; the remaining interval (13 to 100 Hz) was divided into 5 Hz-frequency bins resulting in 21 features per channel. The twelve-band model (or 10 Hz model) started the same with frequency bands delta to alpha, it split the beta frequency range in two (much like the seven frequency band model described earlier) for a low beta (13 to 19 Hz) and a high beta (20 to 30 Hz), the gamma band was then equally split in 10 Hz frequency bins from 30 to 100 Hz for a total of 12 features (or bands) per channel. Refer to the previous section for the seven-band model. The five-band model, the lowest resolution model, consisted simply of the five main neural oscillations: delta, theta, alpha, beta (13-30 Hz) and gamma (30 Hz to cut-off frequency). These PSD feature sets were compared to the 1 Hz frequency bins model and to each other. The last PSD feature model were created to study the influence of cut-off frequency. The gamma band cut-off frequency appears inconsistent in the EEG literature: Buzsaki (2011) uses both 80 Hz and 100 Hz as a gamma band cut-off at multiple occasions in his book on brain oscillations. Other researchers suggest that gamma band activity could extend to 200 Hz (Uhlhaas et al., 2011), while representations of EEG grand average PSD often stop at 45 Hz. Hence, feature sets were extracted with a 100, 80, and 45 Hz cut-off frequency.

Because the N-back task had temporal information from the ERP response available as well as PSDs, additional models were considered for this task inspired by the following study (Brouwer et al., 2012). One model consisted of temporal features from the time-locked epochs: the amplitude of the signal from 0 to 1,000 ms taken every 10 ms, (100 initial features per channel). Lastly, a mixed set of features, dubbed the “fusion” features by Brouwer et al., incorporated the 100 temporal features and 1 Hz interval PSD features for a total of 200 initial features per channel.

The choice of a swLDA classification algorithm was made since it greatly reduced the number of features considered, thereby reducing the risk of over-fitting (Blankertz et al., 2011), important to address considering the high number of features some models have compared to the number of trials. This technique has proven very powerful for EEG single-trial classification (Krusienski et al., 2006). The swLDA was implemented according to specifications from De Vos et al. (2014b). Feature selection was done sequentially and features were only included if they improved class discrimination statistically (*p*_*in*_ < 0.1). For each new feature included, the procedure re-analyzed the current feature pool and removed any feature that had become redundant (*p*_*out*_ > 0.15).

All classifier models in this study were binary. For the arithmetic task, conditions were defined as high and low cognitive workload state. Data from levels 1 and 2 were combined to form the low cognitive workload condition, and levels 4 and 5 were combined to form the high cognitive workload condition. Level 3 was left out to maintain a balanced set of trials per class. This produced a set of over 1250 samples across 2 classes for low vs. high cognitive workload conditions. For the N-back data, high and low working memory conditions were associated with the 2-back and 0-back. This data set held over 700 samples over 2 classes for the high vs. low condition.

The performance of each model was obtained using a ten-fold cross validation test. Before training, the models were divided into 10 sub-groups. Each trial was given a number from 1 to 10, ensuring the same partition of trials or samples was used to evaluate the performance of all the feature sets. The classifiers were trained on all the subgroups except for one, the testing set, which would be used to evaluate the classification accuracy of the trained model. The model then classifies each trial from the testing set using the knowledge it gathered during the training phase. Classification accuracy corresponds to the percentage of trials in the testing set correctly classified as low or high focus by the model. The process was repeated so that each subgroup served as the testing set, yielding ten accuracy results for each model and subject. Reported subject accuracy was the average of these 10 results.

The threshold for above chance-level classification accuracy of single subject data was set using the method in Combrisson and Jerbi (2015), which considers sample size to determine chance-level. Seeing as there were more than 500 samples for each task, in the high vs. low and pairwise binary classification, and that these were 2-class determinations, the chance-level was set at 57% (for a *p*-value of 0.001).

To compare statistical performance of cap and ear-EEG classifiers and assess the influence of different feature extraction and selection methods, a paired t-test was used on the performance metrics. Each model had 10 accuracy results per subject, a *p*-value of 0.001 was considered significant, some average *p*-values were reported when relevant.

## 3. Results

### 3.1. EEG data

#### 3.1.1. N-back task

The N-back task's data epochs were locked to the presentation of a stimuli. This made it possible to study PSD and ERP amplitudes to assess the fluctuations in EEG activity between low and high working memory conditions. The difference between these states was assessed on group data from all subjects and on an individual subject basis to create statistical heatmaps.

**Figure 3:**
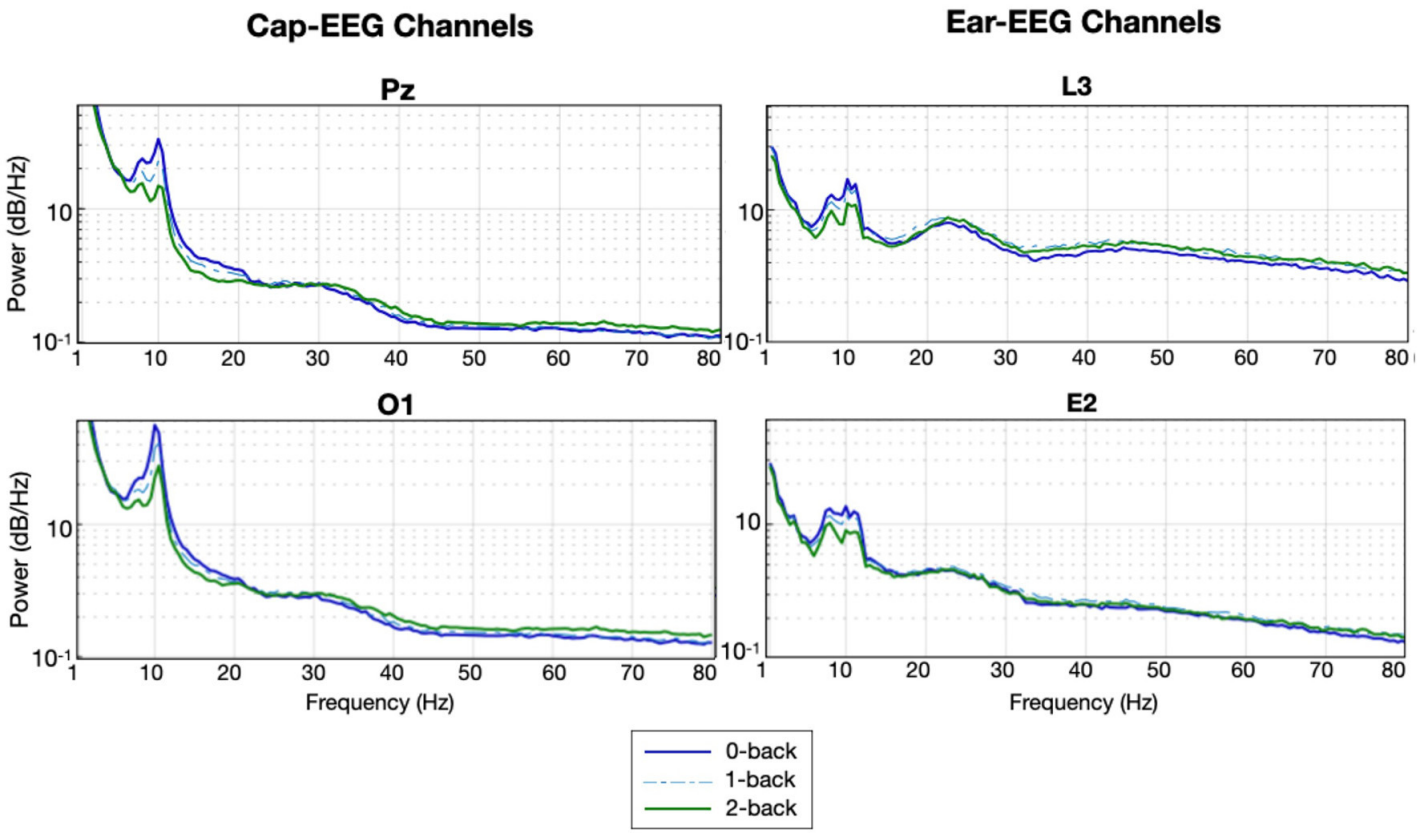
Grand average PSD for the N-back task for selected cap and ear-EEG channels (*N* = 14).

Figure 3 shows the grand average PSD for two cap and two ear-EEG channels for the three levels of the N-back task on a logarithmic scale. The grand average PSD shows three distinct conditions visible with both recording systems, ear and cap, particularly around the alpha peak (8–12 Hz) for all four channels represented. The amplitudes of the alpha peaks are higher for the cap-EEG channels, Pz and O1, compared to the ear-EEG channels. Conditions with higher working memory load exhibited visibly weaker alpha peaks over both recording systems. It also appears that this order, with the least demanding condition on top, the moderate condition in the middle and the more demanding condition at the bottom seems to be the same over the theta range (4–7 Hz) and the lower part of the beta range (below 20 Hz). This holds true for virtually all channels available over both recording systems with a few exceptions where PSD levels were less distinctive. Around 20 Hz and to the end of the frequency range considered, the order of the plot lines gets inverted with the more demanding condition now generating higher PSD values over the high beta range (from 20 to 30 Hz) and gamma range (30 Hz and above).

**Figure 4:**
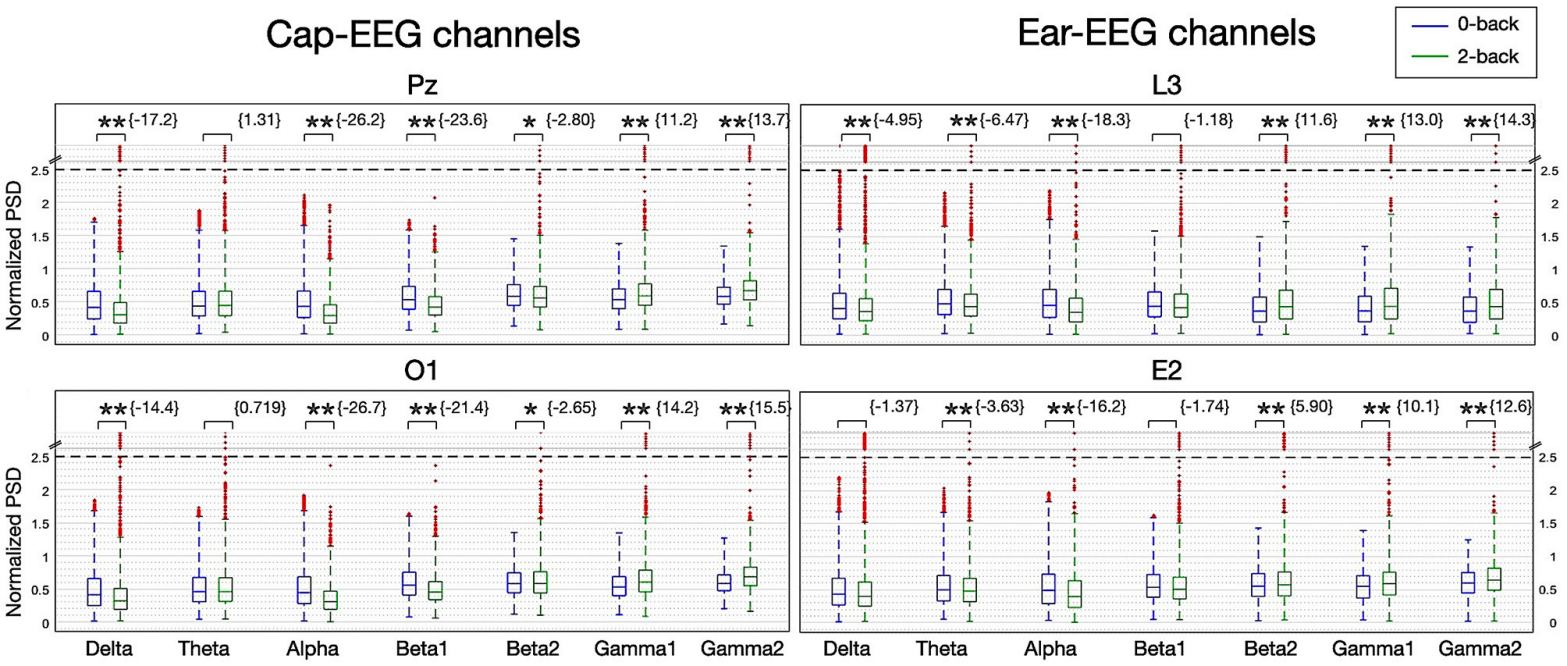
Boxplot of PSD for seven frequency bands for the N-back task for selected cap and ear-EEG channels (*N* = 14; *z-score>± 2.3262 eq. *p* = 0.01, **z-score>± 3.0902 eq. *p* = 0.001).

In Figure 4, the normalized PSD for different neural oscillations for all subjects is represented with boxplots. The range is divided into seven frequency bands: delta, theta, alpha, two beta bands, low beta (beta 1 for 20-30 Hz) and high beta (beta 2 for 20–30 Hz) and two gamma bands, low gamma (gamma 1 30-50 Hz) and high gamma (gamma 2 50–100 Hz). The beta band was split to coincide with the shift seen in the grand averages of Figure 3. The gamma band mid-point between low and high was chosen after it was determined that more splits or different frequency splits than 50 Hz did not have a significant impact on representation or statistical results. In order to visualize power bands from all the frequency ranges in one figure, band power values were normalized at the individual subject level (divided by the 90th percentile for this band power). Normalized values from all subjects were then pooled together and plotted. The data sets have a substantial amount of outliers and many with extreme values. To reduce the number of outliers and improve readability, the interquartile range used for the whiskers of the boxplots is extended to 2.5. Outliers with extreme values are compressed if they are above 2.5 normalized PSD. A dotted line is placed at this upper limit and outlier markers are plotted evenly in the region above it. Z-scores obtained from permutation testing over trials from all subjects are given over each boxplot. The higher the absolute value of the z-score, the more significant the difference between the two mental load conditions. The sign of the z-score indicates the direction of this difference (i.e. positive values indicate the more demanding condition, the 2-back in this case, yields higher PSD values than the less demanding condition, the 0-back).

From Figure 4, it can be noted that most of the distributions are different and highly significantly from their z-scores, for all channels considered. Though the boxplots themselves can sometimes be harder to interpret, particularly with median values, the same trends as with the grand averages (Figure 3) can still be observed over both recording systems. Box outlines, whisker limits and outlier values hold noticeable differentiating information between conditions. That is a tendency for smaller PSD values as mental load increases for the lower frequency bands (under 30 Hz). All channels represented had negative Z-scores for the delta, alpha and beta 1 bands. The alpha bands are the most distinguishable, yielding the highest negative z-scores for every channel considered. While from the beta 2 band onward the PSD is now higher with increasing mental load. All gamma bands z-scores are negative and highly significant and although beta 2's z-score is positive for the cap channels and negative for the ear channels, only the most demanding condition distributions have outliers, with extreme values, for channels over both recording systems for these three highest power bands.

**Figure 5:**
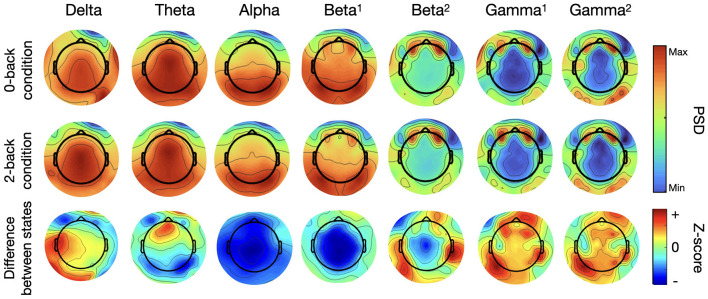
Topographical maps of PSD for seven frequency bands for the N-back task (*N* = 14, first two rows are grand averages, the last is the statistical results of permutation testing between both conditions–positive z-scores (red) indicate an increase in PSD from the 0-back to the 2-back, negative z-scores (blue) indicate a decrease).

Spatial distribution of the PSD over the seven frequency bands can be observed in Figure 5, in which the first two rows show grand average EEG activity for the 0-back and 2-back condition. The last row represents the difference between both conditions by mapping the z-scores obtained through permutation testing for each frequency band at each electrode location. Blue regions represent a decrease in EEG activity with increasing mental load for this neural oscillation; red regions, an increase. The intensity of the color conveys the degree of statistical significance.

The grand average maps for each condition at frequency ranges under 20 Hz - delta to beta 1–concentrate high PSD values over the center of the map with a more posterior focus for the alpha band and the beta 1. Higher frequency range maps show that PSD values are the highest over the ocular regions, due mostly to electro-oculography (EOG) and other muscle-related artefacts. This activity was highlighted by the presence of two dedicated eye electrodes, placed under each eye. Other regions of interest for these higher frequency bands can be seen over the occipital region, increasing seemingly with each neural oscillation range from beta 2 to gamma 2.

When comparing the activity between both working memory conditions, the difference maps show a decrease in activity (represented in blue) for the lower frequency bands. The alpha and beta 1 difference maps are predominantly blue over the entire scalp, while the difference map from the theta band exhibits a decrease in activity in the occipital region that was not visible in the previous maps. Regarding the 3 higher frequency bands, beta 2 (20-30Hz), gamma 1 (30-50Hz) and gamma 2 (50-100Hz), although PSD values were higher over the ocular regions, the differences in PSD distributions across the scalp point to an increase (represented in red) in activity between conditions distributed over multiple regions spanning the entire scalp. Interestingly, regions of most significant statistical difference are concentrated at the back and to the sides of the topographical maps, indicating that the differences between working memory conditions seem to emerge from an increase in EEG activity distributed over the parietal and occipital lobe and not driven by artefacts. These results are in line with our previous findings for the different neural oscillations.

**Figure 6:**
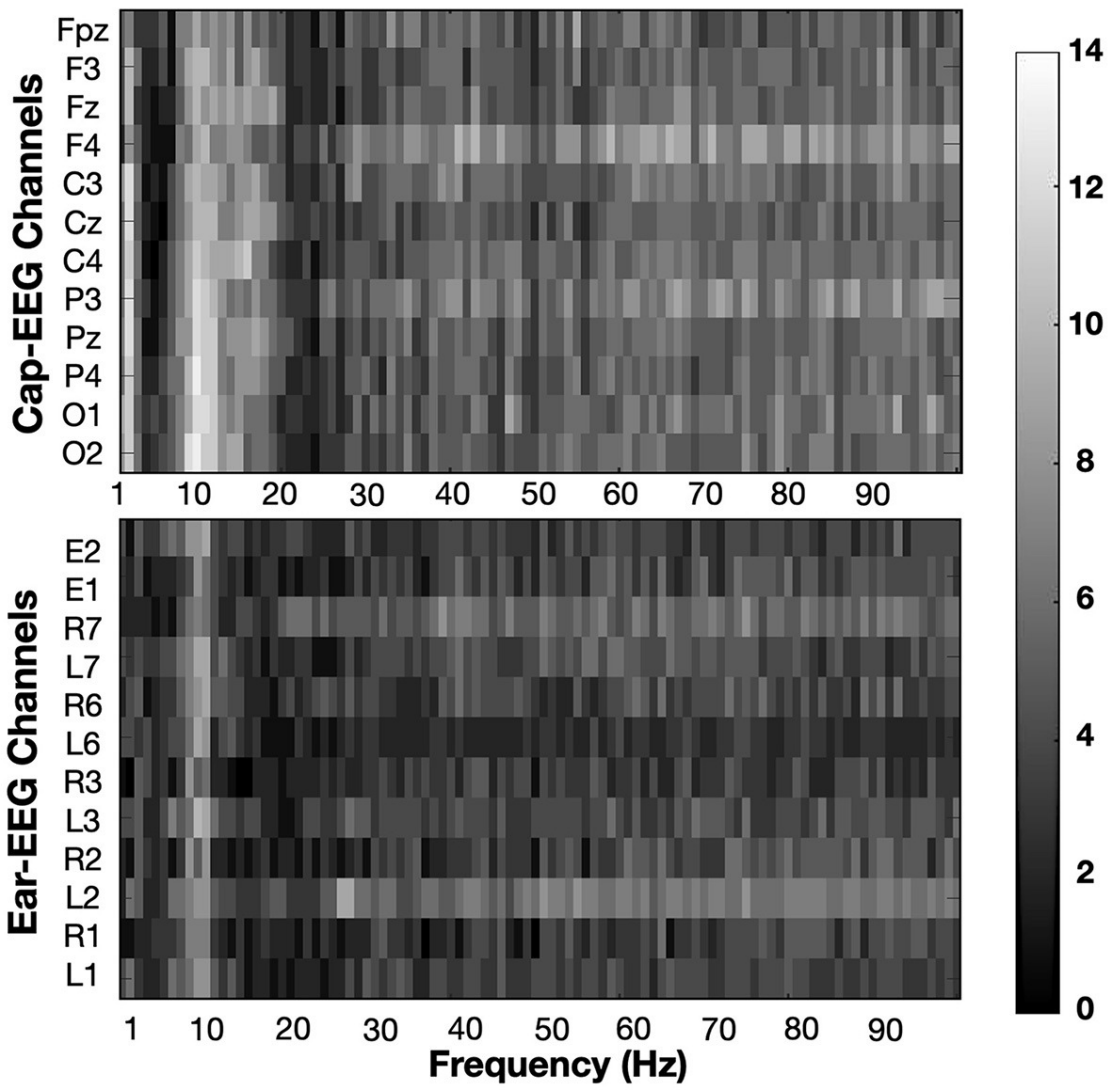
Heatmaps show the sum of subjects (from 0 to 14) for which PSD values are significantly different between working memory condition (0-back vs 2-back). Significance threshold was placed at the subject level at a FDR corrected z-score of ± 2.3262 (equivalent to *p* = 0.01).

Figure 6 shows the differences at an individual subject level. The heatmaps are the result of multiple statistical analyses conducted for each subject and later summed together to highlight which PSD regions and which channels are statistically significant when comparing high and low working memory conditions. At the individual level, subjects exhibited strong differences throughout the power spectrum, which were assessed through permutation testing for each 1 Hz interval bin for individual cap and ear channels. For each subject, every bin was tested and given a binary result, 1 if the difference in PSD was significant at an equivalent *p*-value of 0.01, 0 if it was not significant. Converting the signed z-score to their p-values allowed positive and negative differences to be considered significant instead of canceling their effect when taken in a grand average (Figure 3) or a distribution (Figure 4) approach. Indeed some subjects had inverted effects when considering the same frequency bin and channel. Additionally and to account for the considerable amount of tests per subject (100 bins for 12 channels), *p*-value threshold were adjusted for each subject using the FDR method. Corrected significance threshold actually varied between 0.001 and 0.009 for the cap-EEG channels and from 0.00006 to 0.008 for the ear-EEG channels. Each rectangle of the statistical heatmap represents the sum of subjects for which this PSD bin was significantly different at this channel. Twelve channels were represented for each recording system, the same as were later used for classification. The alpha peak range, centered around 10 Hz, is visibly lighter across all channels considered, for both recording systems. Some other areas are more significant for the cap-EEG channels: the delta range (1–3 Hz) and the frequency bins right after the alpha peak where we can distinguish a slow gradient of color throughout the low beta range from 12 to 20 Hz ending with a darker, less differentiated EEG activity between 20 and 30 Hz. For the gamma range, beyond 30 Hz, both recording systems indicate that a high number of individual subjects exhibit significant differences over this frequency range.

**Figure 7:**
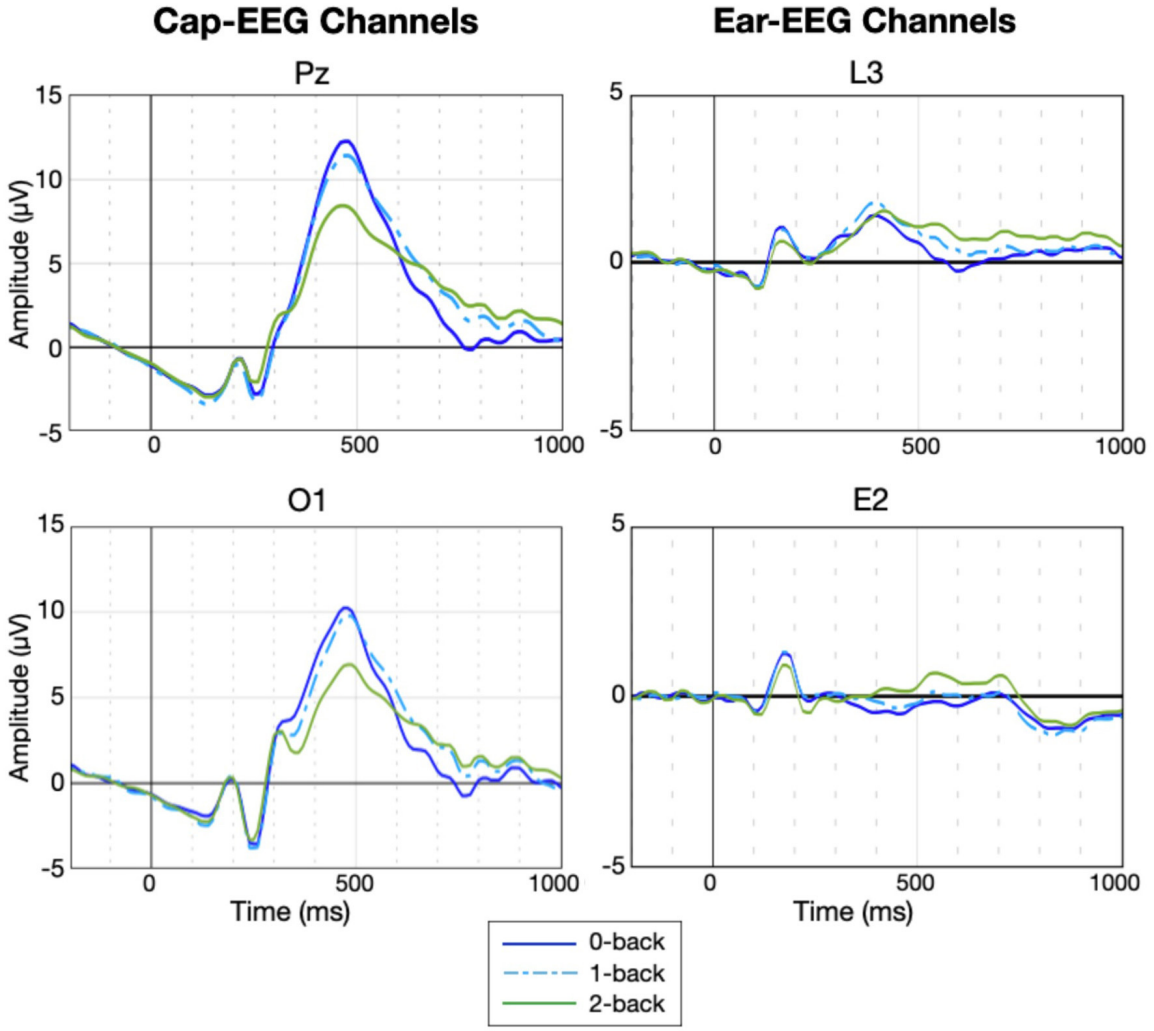
Grand average ERP for the N-back task for selected cap and ear-EEG channels (*N* = 14).

**Figure 8:**
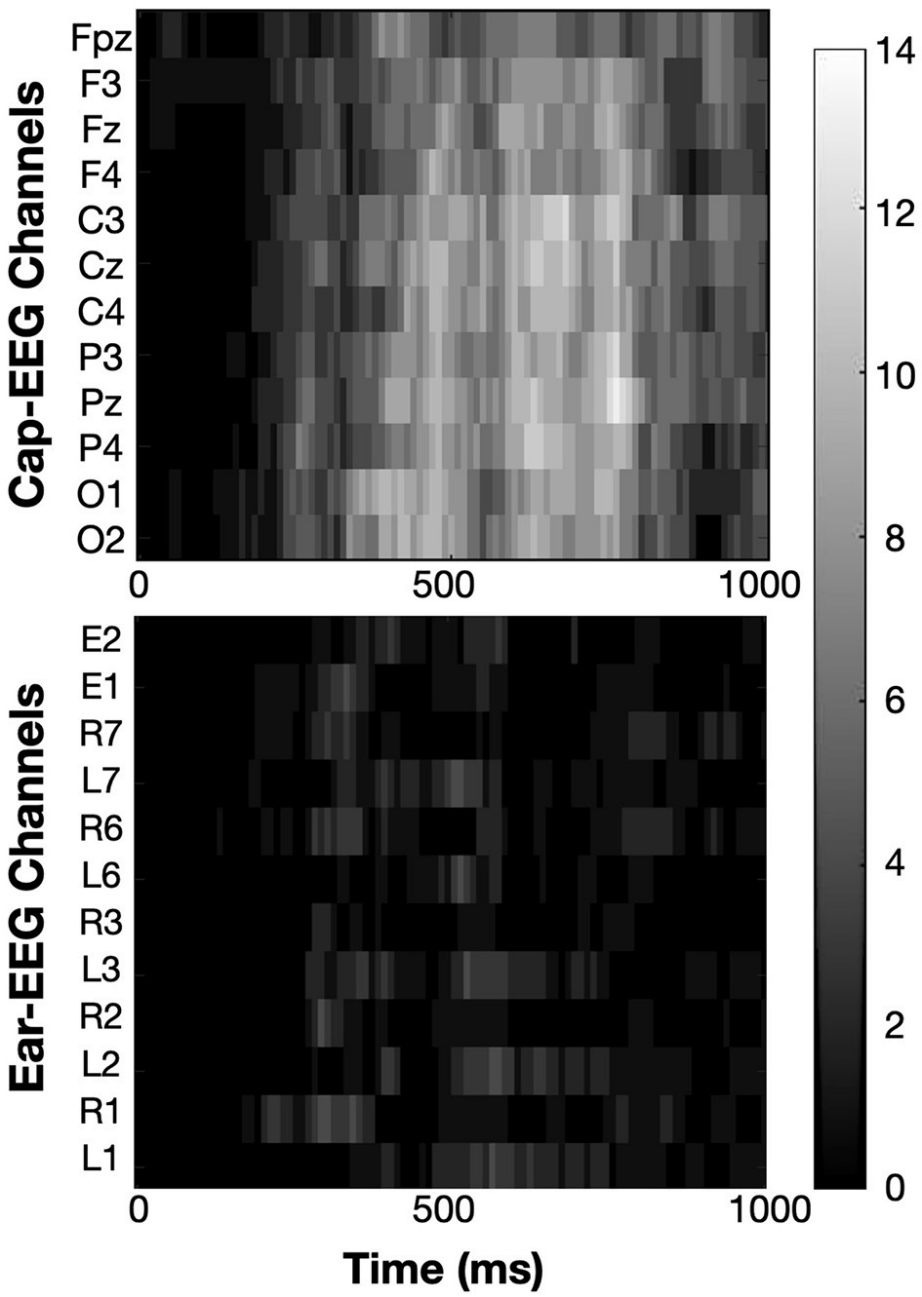
Heatmaps show the sum of subjects (from 0 to 14) for which ERP amplitudes are significantly different between working memory condition (0-back vs 2-back). Significance threshold was placed at the subject level at a FDR corrected z-score of ± 2.3262 (equivalent to *p* = 0.01).

Figure 7 shows the grand average ERP waveforms generated by the N-back task for the same electrodes as the PSD figures. Cap-EEG channels showed peaks at much higher amplitudes than ear-EEG channels. Given that the ear-EEG ERP is considerably noisier, a 4-point moving average was applied to smooth the data. For the cap-EEG channels, a distinct P300 response started around 250 ms and peaked at around 450-500 ms after stimulus onset. The P300 peak was visibly higher for lower working memory conditions and return to baseline was faster. The ear-EEG waveforms are not as clear. The around-the-ear electrode L3 highlights a visible P300 response starting a bit earlier than for the cap-EEG channels but the differences between conditions are not clear at the P300 peak level, return to baseline seems to be faster again for lower working memory condition. The in-ear data grand average ERP shows a clear P100 response, present in all the considered channels, but the P300 response is not visible. This difference between recording equipment, between the cap-EEG data's recognizable P300 peak and the ear-EEG data's much less discernible ERP waveforms is supported by the statistical heatmap for the temporal data analysis, Figure 8. This heatmap was obtained using the same method as for Figure 6, comparing at the individual subject level, PSD for low (0-back) and high working memory (2-back) conditions at each time-point (10ms intervals) and for each of the twelve channel subsets. Significance threshold at the subject level was place at an equivalent of *p* = 0.01 and corrected for multiple comparisons using the FDR method, actual p-value thresholds varied between 0.002 to 0.005 for the cap-EEG channels and from 0.000003 to 0.0014 for the ear-EEG channels. Each rectangle of the heatmaps represents the sum of subjects for which this amplitude is significantly different at this channel. The cap-EEG channels showed very bright regions around 500 ms, corresponding to the peak of the P300 response seen in the grand average waveforms. However, the ear-EEG channels, particularly the in-ear channels (E1 and E2) don't show as much shared differences in the temporal domain. Some outer-ear channels were still able to detect significant changes in amplitude in the range of the P300 response for a majority of subjects.

#### 3.1.2. Arithmetic task

For this task, the PSD was taken from 2-second epochs taken at regular interval, no time domain analysis was performed. To assess the fluctuations in EEG activity between a high and a low cognitive workload, subjects were asked to solve arithmetic problems with varying degrees of difficulty. The differences were assessed on group data from ten subjects and an individual subject basis to create the heatmaps.

**Figure 9:**
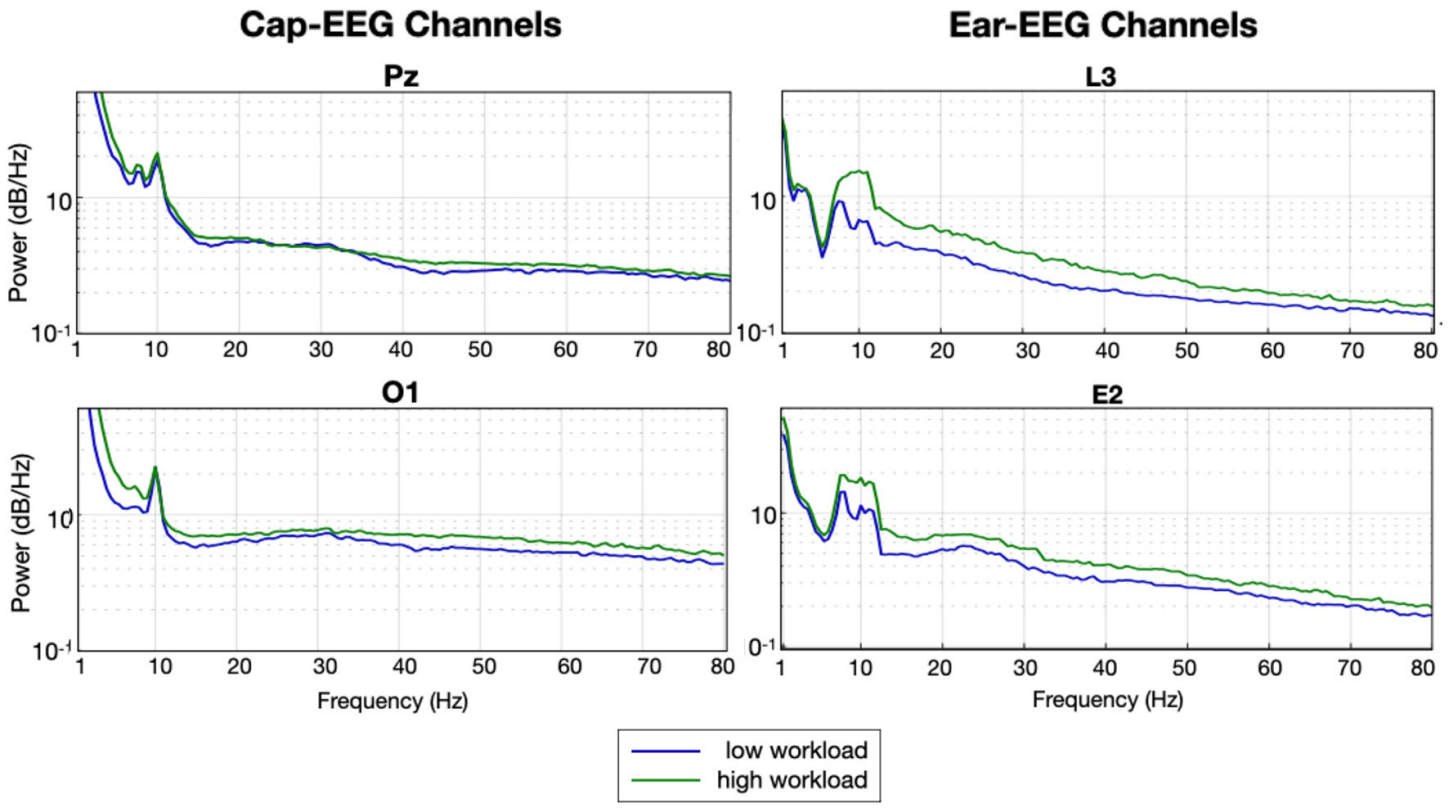
Grand average PSD for the arithmetic task for selected cap and ear-EEG channels (*N* = 10).

Figure 9 shows the grand average PSD for the arithmetic task. For this task also the different mental load conditions can be differentiated on these plots for both recording systems. The alpha peak was harder to differentiate between low and high workload conditions for the cap-EEG than for the ear-EEG. For this task and for both recording equipment, the more demanding condition appear to generate higher PSD than the less demanding one, over the entire frequency range considered. The grand average ear-EEG PSD difference is quite striking from the alpha range forward. The gamma band PSD for the cap-EEG data was also distinctly higher for the more demanding workload condition.

**Figure 10:**
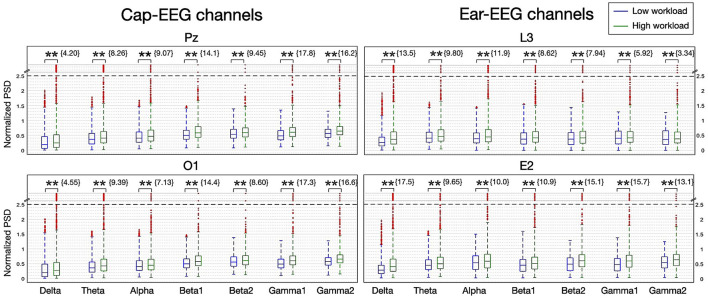
Boxplot of PSD for seven frequency bands for the arithmetic task for selected cap and ear-EEG channels (N=10; *z-score > ± 2.3262 - eq. *p* = 0.01, **z-score > ± 3.0902 - eq. *p* = 0.001).

The boxplots of Figure 10 represent the distribution of PSD for all subjects for the seven frequency bands. The data was analyzed in the same manner as for the N-back task, the frequency bands were the same, the normalization procedure and statistical approach were also the same (refer to Figure 6). The boxplots confirm and highlight that the more demanding cognitive workload condition's PSD is statistically higher than the less demanding condition for all frequency bands and for all channels considered from both recording equipment, indeed all z-scores obtained from permutation testing are highly significant and they are all positive. Despite the normalization of the data and the increase in the percentage of distribution included within the whiskers of the plots, there is still a large number of outliers for this task, like for the one before. For this task however, extreme value outliers are only found for the more demanding condition, and the higher the frequency bands, the more this difference in outlier presence differs between the conditions. From beta 2 to the gamma ranges, the distributions for the low workload condition show no outliers for all channels considered over both recording systems while the high workload condition distributions exhibit many and always with some extreme values. These non-uniform distributions could suggest that the pooled data from all subjects might need better processing methods to harmonize results and lower variance.

**Figure 11:**
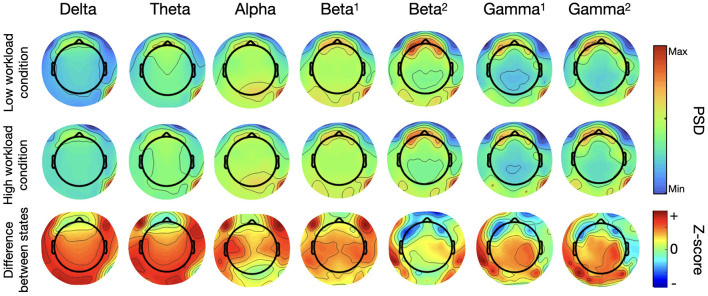
Topographical maps of PSD for seven frequency bands for the arithmetic task (N=10, first two rows are grand averages, the last is the statistical results of permutation testing between both conditions–positive z-scores (red) indicate an increase in PSD from the 0-back to the 2-back, negative z-scores (blue) indicate a decrease).

The topographical mapping of the seven frequency bands is shown in Figure 11. The maps show the grand average PSD at each channel location for both conditions and the difference between conditions by way of projecting the results of a statistical analysis at each electrode location (cf to Figure 5 for more details as the procedure is the same). The grand average maps are not so informative, there seems to be a strong unilateral posterior component present in all frequency ranges and strong activation over the ocular region as well, due to EOG artefacts, for the beta and gamma bands. Other important regions and differences between states are not easily identifiable in these rows but they come into focus with the difference maps. Mostly the difference maps show an increase in EEG activity (seen in red) throughout the frequency range considered. The posterior region, over the occipital lobe, seems to be the most significant when considering the highest (high beta and both gammas) and the lowest (delta and theta) frequency bands, indicating again that differences between conditions is emerging from actual EEG activity. Maps from the middle of the frequency range, alpha and low beta, show more lateralized regions of interest over the parietal and frontal lobe. For this task, EOG activity seems to have a localized differentiated effect between mental load conditions, particularly for the higher frequency bands where artifact activity decreases.

**Figure 12:**
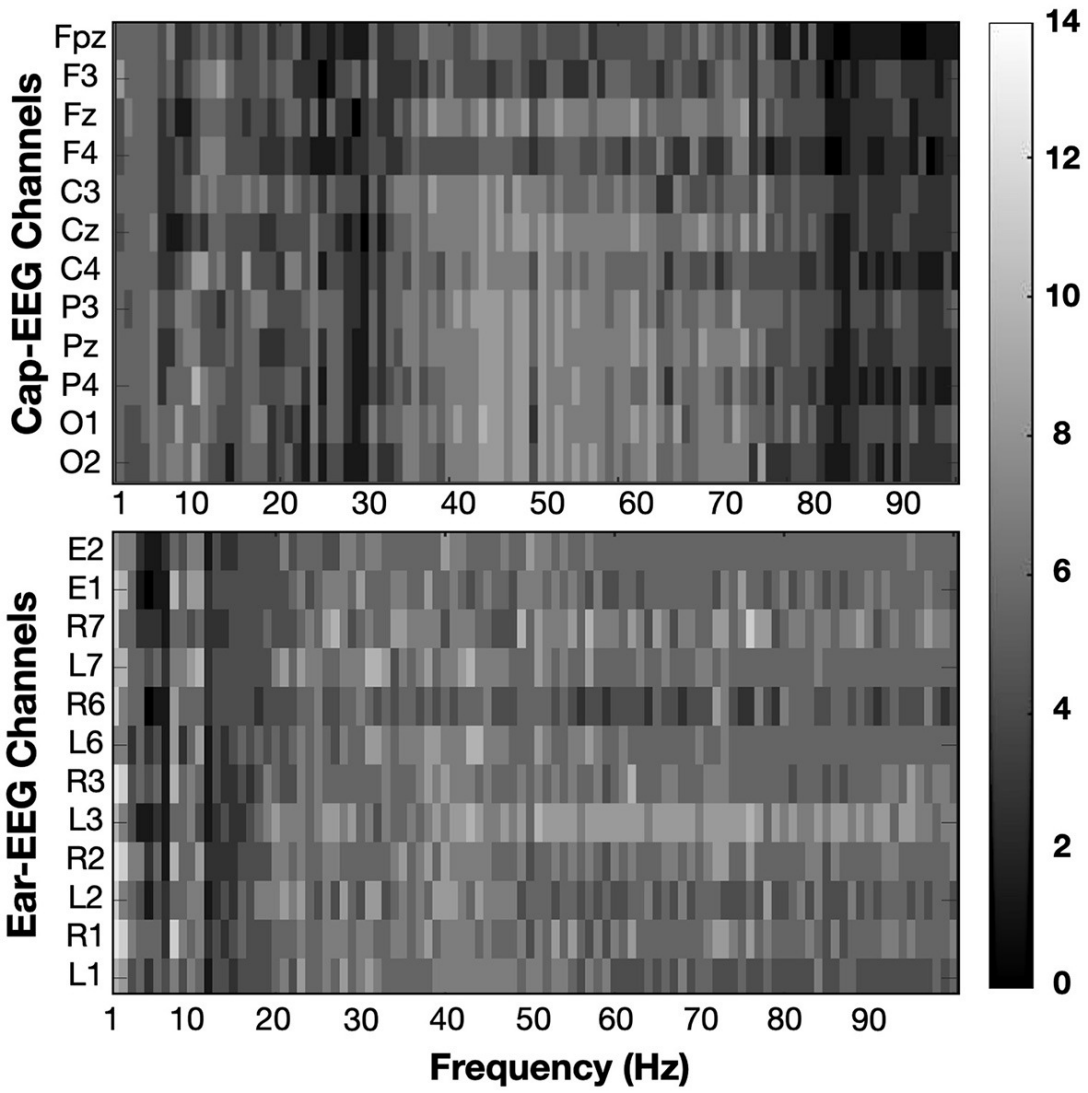
Heatmaps showing the sum of subjects (from 0 to 10) for which PSD values are significantly different between cognitive workload conditions (low vs. high). Significance threshold was placed at the subject level at a FDR corrected z-score of ± 2.3262 (equivalent to *p* = 0.01).

Figure 12 highlights the number of subjects showing a significant difference between low and high cognitive workload conditions in PSD from 1 to 100 Hz for the same twelve channels subset for each recording equipment as before. The approach is the same as for the N-back task, refer to Figure 6 for more details. Significance was calculated through permutation testing and the resulting statistical scores adjusted using the FDR method for multiple comparison correction. Significance threshold was put at *p* = 0.01, adjusted *p*-values varied for each subject ranging from 0.00011 to 0.0072 with an average of 0.0031 for cap-EEG channels and from 0.00017 to 0.0085 with an average of 0.0037 for ear-EEG channels. The alpha range for this task appear less differentiated across conditions than for the last. Some channels have some very bright bins in this interval, but for the cap-EEG data at least, there is no clear band across all channels. The ear-EEG data has a clearer indication of strong differences in the alpha range flanked by a very dark region over the theta range (4–7Hz) and a darker region just following it. This is not to say that there were less significant differences in the PSD for this task, very light areas are found throughout the heatmaps. They were more largely distributed across the higher frequency ranges, mostly beyond 20 Hz for the ear-EEG data and beyond 30 Hz for the cap-EEG data. Lastly, the cap-EEG data appears to be less significant for individual subjects beyond 80 Hz although this tendency was not observed in the ear-EEG data.

### 3.2. Classification results

#### 3.2.1. N-back task

**Figure 13:**
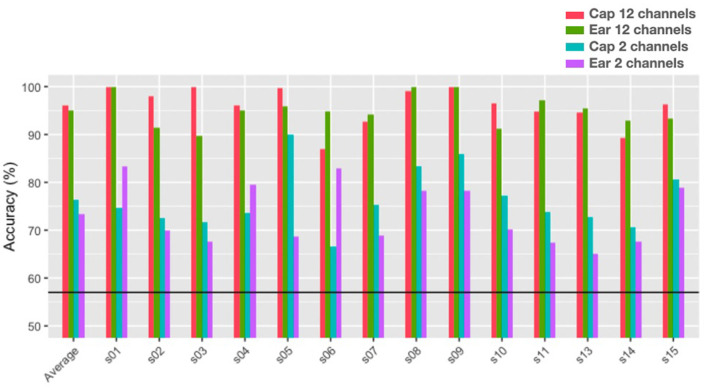
Single-Trial classification accuracies for the N-back task with a 1 Hz interval bins and a 1–100 Hz frequency range (the black line represents chance level at 57%).

Figure 13 shows individual subject's single-trial classification accuracies obtained using an swLDA classifier to predict the class of individual trials between the high (2-back) and low (0-back) working memory conditions. The algorithm first performed a feature selection step, the step-wise linear regression which kept only the more statistically differentiated features. Then the model was trained on a portion of the trials and tested on the remaining trials using only the selected features. Accuracy results show the percentage of correctly classified trials within the testing sets using a 10-fold cross-validation scheme. For each subject, the average of these 10 accuracies was reported and the average over all subjects is plotted in the same figure. The features used were extracted from the 1 Hz spectral feature range model. This model consists of PSD extracted from each epoch or trial from 1 to 100 Hz in 1 Hz interval bins, much like the statistical heatmap data (Figure 6). The heatmap figure represented the same channels considered for classification, the lighter regions of the heatmaps showed where the significant features are concentrated and there is a strong overlap between these regions and the selected features of the classifier. They are the features that help differentiate between working memory loads. This feature model started with 100 features (PSDs) per channel, 1,200 for the 12 channel model, which represents a higher number than the number of trials (768). This creates issues of dimensionality that are problematic for classification purposes. The ill-advised feature to sample ratio was mitigated through the feature selection regression. On average, over fourteen subjects, the final number of features kept for this model went from 1,200 for the twelve channel models to 306 for the cap-EEG data and 285 for the ear-EEG data. For the two channel models, the initial number of features was 200 and the number of features used for classification was on average 35 for the cap-EEG data and 34 for the ear-EEG data.

Classification results of Figure 13 show that all subjects achieved significant above-chance level performance for both recording equipment, for the twelve and the two-channel models. For the twelve channel models, average accuracy was 96% for the cap-EEG and 95% for the ear-EEG;. For the two-channel models, average accuracy was 76% for the cap-EEG and 74% for the in-ear EEG. It might appear that cap-EEG data yielded higher classification accuracies than ear-EEG data but these differences were not found to be statistically significant with a paired t-test comparing the 10 fold classification accuracies of all subjects and a *p* = 0.001 threshold (*p* = 0.034 for the twelve channels and *p* = 0.230 for the two channels). On an individual subject level, it is interesting to note that some of the two channel ear-EEG models which represent only dry in-ear electrodes outperform greatly the occipital channels of the cap-EEG models, it is the case of subject 1 and 6 for example and subject 4 to a lesser extent.

For the N-back task, time domain features were also available. Table 1 provides the classification accuracy for different feature modalities: spectral, temporal and mixed. Both cap and ear-EEG data were considered for both channel models. The spectral 1 Hz interval bins feature and the temporal 1,000 ms (in 10 ms interval) features started out with the same number of initial features, 100. The mixed or “fusion” model had 200 initial features per channels. The results show that the spectral domain features outperformed the temporal domain features and that the fusion model outperformed both of them. This is statistically significant for both channel subsets across both recording systems with p-values all under 10^-7^. While the performances for the spectral range models did not differ statistically between recording equipment. The performances of the classifier for the temporal and fusion models were all significantly better for the cap-EEG data than the ear-EEG data with p-values all under 10^-4^ for both channel subsets and both feature model.

**Table 1:** N-back task single-trial classification results for different feature modalities (in grey is the final number of features kept by each classifier over the initial number of features available).

	**12 channels**		**2 channels**	
	**cap-EEG**	**ear-EEG**	**cap-EEG**	**ear-EEG**
Range 1 to 100 Hz 1 Hz steps	96 %	95 %	76	73 %
	(306/1,200)	(285/1200)	(35/200 %)	(34/200)
Temporal 1 to 1000 ms	86 %	76 %	72 %	64 %
	(76/1,200)	(57/1200)	(17/200)	(16/200)
Fusion: temporal and 1 to 100 Hz	100 %	99 %	81 %	77 %
	(564/2,400)	(592/2400)	(48/400)	(52/400)

When considering the final number of features for each model, it can be noted that the final number of temporal features was a lot less than the final number of spectral features, about 80 % less for the 12 channel models and close to 50 % for the 2 channel model. The final number of mixed features selected by the step-wise linear regression was unexpectedly higher than the addition of those different modality features for the twelve channel configuration, 37% more features on average. This could explain the abnormally high classification accuracies of the fusion model for the twelve channel models, probably brought on by over-fitting. This was not the case for the 2 channel model.

#### 3.2.2. Arithmetic task

**Figure 14:**
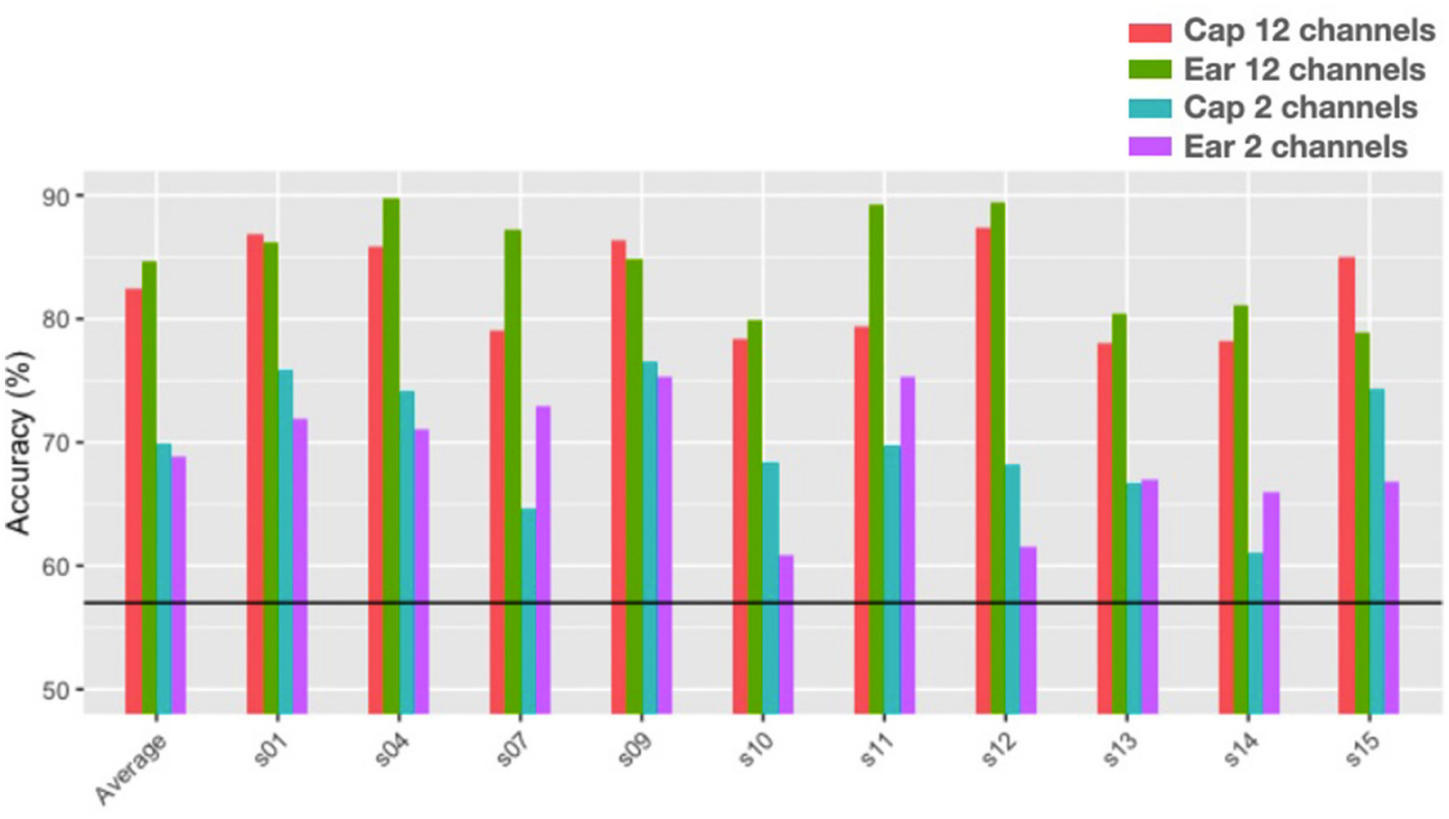
Single-trial classification accuracies for the arithmetic task with a 1 Hz interval bins and a 1–100 Hz frequency range (the black line represents chance level at 57%).

Figure 14 shows the single subject classification accuracy for the same model as in Figure 13 for the arithmetic task (1 Hz interval frequency range model from 1 to 100 Hz) for each recording system and for a twelve and two-channel feature set. The initial number of features was again undesirably close to the number of trials, around 1,392 compared to 1,200 initial features for the twelve-channel model. Again, the step-wise linear regression step helped reduce the number of features considered for classification to 139 for the twelve-channel cap-EEG, 171 for the twelve-channel cap-EEG data, 34 for the two-channel cap-EEG and 36 for the two-channel ear-EEG. The figure shows that all subjects performed better than chance-level for the twelve-channel feature set with an average of 81% for cap-EEG, 84% for ear-EEG. For the two-channel feature set, the average was 70% for the cap-EEG and 69% for the in-ear EEG. On average, it seems that the ear-EEG features outperformed the cap-EEG ones for the twelve channel model but not for the two channel model. However, none of these differences are significant at an alpha of 0.001 although the twelve channel ear-EEG model comes close to this threshold (*p*= 0.0013 for the twelve-channel, *p*= 0.309 for the two-channel). Some individual subjects show better performances for the in-ear EEG data compared to the cap in this task as well, such is the case for subject 7 and 11 for example.

#### 3.2.3. Spectral feature model comparison

**Figure 15:**
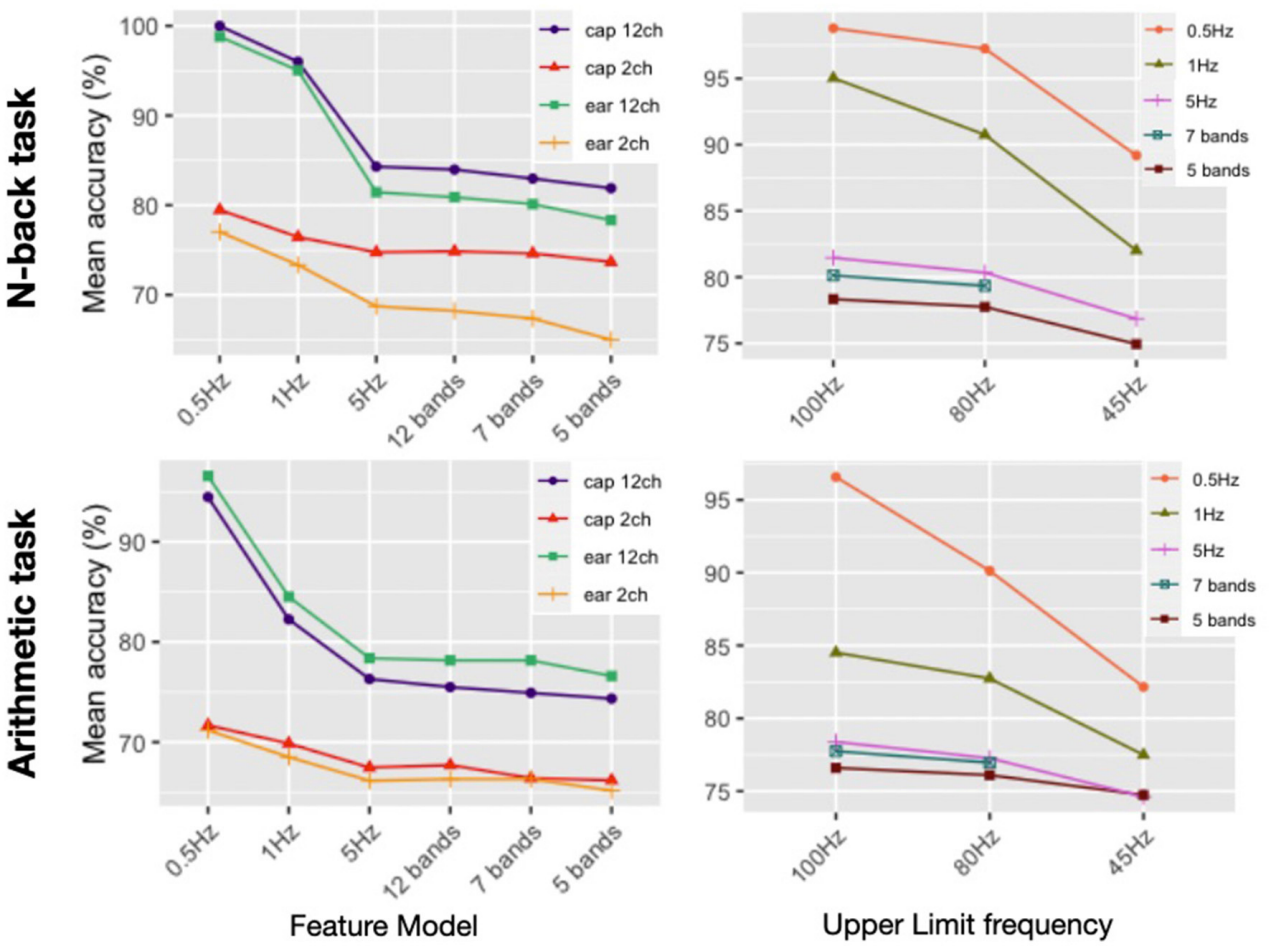
Average of individual subject's classification accuracies for different spectral resolution and frequency range cut-off for the N-back (*N* = 14) and arithmetic task (*N* = 10).

Figure 15 is useful to assess the influence of certain feature parameters over classification accuracies. The same tendencies can be observed for both tasks: on the left, a decrease in classification accuracy with decreasing spectral resolution; on the right, a decrease in classification accuracy with decreasing frequency cut-off for the spectral range considered. The feature models in the left plot all have a frequency cut-off of 100 Hz. The accuracy results from the right plots are those of the twelve channel ear-EEG model, the tendencies were the same for other channel subsets. The feature sets are described in detail in Section 2.5. They are labeled as 0.5 Hz for 0.5 Hz-interval bins (200 initial features per channel); 1 Hz for the 1 Hz-interval bins (100 initial features per channel); the 5 Hz model corresponds to bands of 5 Hz or less (21 initial features per channel); and finally the band models have the same number of initial features per channel as their band number.

Statistically for the N-back task, each decrease in resolution from 0.5 Hz down to 5 Hz did yield a significantly lower reported classification accuracy average for all channel models. After that resolution, drops in accuracy are not significant (alpha 0.001) from one model to the next until reaching the 5 bands model where all four channel models perform statistically worse than all other models considered. The drop in accuracy and subsequent stagnation can be linked to the number of features in the final models. For the twelve channel models the final number of features is about 540 for the 0.5 Hz models and just under 300 for the 1 Hz model. After this, it drops to below 50 features for the 5 Hz and 17 for the 5 band models. The two channel models only have 5 or 6 final features considered by the classifier yet they still manage to yield accuracies over chance-level (73% for the cap-EEG and 65% for the ear-EEG).

For the arithmetic task, statistical analysis results are quite similar. Significant drops in accuracy can be found from 0.5 to 1 Hz and then again from 1Hz to 5Hz for all four channel. After this, no significant drop from one model to the next is recorded until the 5 band model which performs significantly worse than the 5 Hz model but only for the twelve channel models.

The right side plots show that classification accuracy decreases when the gamma cut-off frequency is lowered. The seven band model stops at 80 Hz because with a 45 Hz cut-off frequency, it consisted of the same features as the 5 Hz model. All reported average accuracy results plotted for both tasks and for the 5 feature models were lower when the cut-off frequency dropped from 100 to 80 Hz and from 80 to 45 Hz. However, not all decreases were statistically significant. Lowering the cut-off to 45 Hz was significant for both tasks for the three range models considered (0.5, 1, and 5 Hz); while the drop in accuracy going to a 80 Hz cut-off frequency was statistically significant for the 0.5Hz and the 1Hz model when considering the N-back task; and for the 1Hz model for the arithmetic task.

## 4. Discussion

Future BCIs have the potential to change how we interact with technology, assuming they can be used outside of controlled environments. Besides comfort and aesthetics, the signal must be of sufficient quality if one is to reliably decode different mental states. Mobile EEG recording equipment with proven signal quality is already available (Debener et al., 2012; De Vos et al., 2014a). In this study, we investigated the potential of ear-EEG to monitor focus, a concept that overlaps with notions such as concentration, attention, engagement and intensity of a task. Tasks were chosen to represent different features of focus, namely working memory and cognitive workload. The goal in both of these tasks was to differentiate between low and high mental demand to ascertain differences in EEG activity that could be indicative the level of focus. We compared the quality of neural oscillation recordings of a mobile ear-EEG recording system to that of a conventional 84-channel wet cap-EEG wired system, used concurrently. The ear-EEG electrodes were wet around-the-ear electrodes (cEEGrids) and dry in-ear electrodes (TIPtrodes^TM^).

When comparing the cap-EEG data obtained with existing studies using similar paradigms (Rebsamen et al., 2011; Brouwer et al., 2012), our results were comparable. For the N-back task, we found a strong and significant alpha peak with its power inversely proportional to the working memory load (Figure 3). The peak was visible in both cap and ear channels, although cap channel power was stronger for this interval. In the higher frequency ranges, above 30 Hz, it seemed the PSD order for both recording equipment inverted with the higher demand translating into higher PSD levels over high beta and gamma bands. Over this higher frequency range, ear channels recorded slightly stronger PSD signals. Overall, these grand average power spectrum seem to offer information of comparable quality across both recording systems as further evidenced by Figure 4. This next figure highlights the differences in distribution of all frequency ranges in more compelling detail, through its representation of the PSD distributions but also thanks to the statistical results it features. The results of the boxplots establish that for this task, EEG activity elicited from a higher working memory demand translates into statistically lower PSD for frequency ranges under 20 Hz; from delta to alpha. Above 30 Hz, higher working memory demand translates to a higher PSD. The transition between this inversion occurs over the beta range from 12 to 30 Hz where it does fluctuate between channels and range considered. Cap channels are quite effective at showing differences in the lower beta range, behaving much like the alpha range distribution: lower medians for the more demanding condition and less outlier values. For the higher beta range, the around-the-ear channel show a statistically more significant and visually more compelling difference where the more demanding condition show a higher distribution and more outliers. The dry in-ear EEG channels' results seemed less conclusive when considering the grand averages, particularly when it came to the higher frequency bands, but the differences evidenced in the boxplots were still very significant for the gamma frequency range.

The topographical maps show how these differences in PSD were distributed spatially. They are consistent with the previous results, upholding the choice of these selected channels to represent EEG activity for this recording system. The shift between the lower frequency ranges which were predominantly blue, indicative of a decrease in activity to maps with a lot more red, indicative of an increase in EEG activity, for the higher frequency range is complemented with more information on the spatial origin of these differences in PSD. The higher frequency bands (high beta and gamma oscillations), residual artifacts of eye movement clearly contaminated the PSD topographical maps. However, it was shown that activity in this ocular region is not where the differences between working memory load are the strongest. For these higher frequency bands, higher working memory load had the strongest statistical significance over posterior regions of the maps. Visible clusters of heightened activity can be seen over the temporal lobes reaching more towards the occipital region. This lets us surmise that although PSD in the high beta and gamma ranges incorporates residual muscle activity which contributed unwittingly to the results, the differences in mental states are in fact driven by activity emanating from cortical areas. This was important to show particularly for the gamma range where EOG activity is known to persist even after appropriate data pre-processing and can be underestimated when using a nose reference (Yuval-Greenberg et al., 2008).

Heatmaps of Figure 6 have shown that differences at the individual subject level are strong for the alpha range and for the higher frequency bands (30Hz and up), sometimes stronger than grand average or cumulative distribution differences. Boxplot representation showed that there is a high degree of variance in data sets obtained from multiple subjects and that the medians are not the best tool to assess differences between these populations. Heatmaps considered individual differences, which were sometimes of opposite signs, to assess which frequency ranges were the most indicative of change in EEG activity between mental load conditions.

The classification accuracy results for this task obtained with a spectral range feature set from 1 to 100 Hz in 1 Hz intervals were well above chance-level, that is, over 95% for the twelve-channel models and over 70% for the two-channel models. The working memory condition could reliably be decoded from both cap and ear-EEG signals as well as in-ear EEG. Cap and ear EEG recording equipment produced comparable results and although the cap-EEG feature sets often had a few percentage points over ear-EEG feature sets, this difference was not significant. This was all the more impressive given that the twelve-channel cap-EEG electrodes were placed around the entire head versus the ear-EEG electrodes closer together and condensed in two much narrower regions around and inside the ears. The two-channel model's comparable performances were noteworthy particularly since we compared occipital wet electrodes to in-ear dry electrodes for a task with visual stimuli. On an individual subject basis, none of the recording equipment was consistently better than the other. A better result with the twelve-channel cap-EEG feature model did not mean that the two-channel cap-EEG would perform better and vice versa.

For the arithmetic task, again we observed clear differences between mental load conditions, however these were not always the same differences as what we saw for the N-back task. The alpha range provided a visible peak and could be used to distinguish between conditions but the difference was mostly for the ear-EEG channels this time and said difference was in the opposite direction than for the other task. The cap-EEG channels grand average representation of PSD (Figure 3) did not provide a clear differentiation between workload levels at this frequency range, although significant differences were confirmed with the boxplots (Figure 4). This is in line with the concept of internalized attention vs. externalized attention (Magosso et al., 2019). For this task, it was observed that with a higher workload came a higher PSD for the entire frequency range considered for both recording equipment. Only the high beta range (20 to 30 Hz) showed areas of overlap between conditions and for cap channels only. Other than this, the low workload conditions had systematically and significantly lower PSD at every frequency range considered. For a majority of the bands, the standard deviation for the low workload condition was also much smaller than the high workload condition. Although the differences in EEG activity for the lower frequency bands (under 20 Hz) were not what was observed for the N-back task, the gamma band distributions had important similarities between the two tasks. When considering the topographical distribution of PSD for each neural oscillation (Figure 5), the difference maps all showed large areas with positive z-scores, which translates to stronger PSD with higher workload demand. The regions where these differences were the strongest and most significant were distributed over several cortical regions according to the frequency range considered. The alpha and lower beta band's PSD changes were concentrated over the temporal region which could be an indication as to why the ear-EEG channels performed better at differentiating activity in that range. A phenomenon confirmed again in the statistical heatmap of Figure 12, where the alpha band was visible for the ear but not so much for the cap-EEG channels. Regarding the higher frequency range maps, similar conclusions could be drawn for both tasks for high beta and both gamma bands. Specifically, grand average workload condition maps pointed to the ocular region, indicative of muscle artefacts, but the most significant areas when considering differences between conditions pointed to the parietal and occipital regions consistent with actual EEG activity. Some clusters were also found closer to the temporal regions for the beta bands. These considerations are important since the features used for classification are selected based on their statistical difference between conditions. Conclusions of the topographical analyses therefore support the argument that the features used for classification are in fact related to actual brain activity dynamics. The classification accuracy results for this task, obtained with the same range model as before, were again well above chance-level, 80% and more for the twelve channel models and close to 70% for the two channel models. The cognitive workload could be decoded from both cap and ear-EEG signals, around and inside the ear. Statistical analysis of both task, did reveal that for this task, the ear-EEG channel sets performed slightly better than the cap-EEG channels for some subjects over both channel models, twelve and two.

We conclude that EEG spectral features recorded from inside and around the ear can reliably assess different focus levels may it be related to working memory or cognitive workload features. These features systematically allow for significantly above-chance level classification accuracy between all levels of difficulty. Therefore, it would be feasible to develop a concentration or focus-monitoring BCI in the form of an earpiece.

The next steps for this line of research will be to test these conclusions under less restrictive conditions, outside of a controlled environment and with more diverse and complex tasks. Such data obtained from more realistic focus conditions will obviously be noisier, and will be harder to appropriately label for the purpose of training new classification algorithms. On the other hand, the tasks presented for this study might have been biased or misrepresented the focused state of individual subjects. The degree to which the participant's might have felt involved in these repetitive tasks throughout the recording and the perceived difficulty of the task could vary as several subjects did comment on the tediousness of the N-back task in particular. Recording participants in more realistic focus scenarios might actually provide better, more consistent differences across PSD features between tasks and between participants. The nature of the tasks will most likely have an impact on which spectral ranges are best to characterize focus. As we have seen here, the alpha frequency although very effective to distinguish between working memory conditions and between cognitive workload conditions behaves in a completely opposite fashion from one task to the other. It might be too task dependent to be useful in this context. Higher frequencies in the beta and gamma bands, although mixed with muscular activity, might procure more consistent markers of focus across a variety of tasks.

This study showed grand average EEG results and distributions from all participants but reported classification accuracies for within-participant trial data. This choice was supported by Aroudi et al. (2016)'s results on attention decoding, which showed that given sufficient individual data, individual models will outperform the group model given that inter-subject variance is always larger than variance within individuals for this type of data. Nevertheless, according to the type of application considered it might still make sense to try and improve group model classification. Going back to the boxplot from both tasks, distributions of PSD values were non-uniform, skewed and contained a lot of outliers. The medians were often very close in value while the spread and reach of the distributions were in fact very different. This might be related to the high variance across subjects which could be improved through better standardization methods when comparing PSD values. Spectral selection, filtering and the use of band ratios seem like interesting avenues to address this (Berka et al., 2007). This will most likely imply further research on feature extraction procedures and preprocessing specifications that can have a great impact on classification accuracies (Farquhar and Hill, 2013). The choices made, that led to the specific features used for this study, included montage selection (electrode discrimination from eighty-four to twelve for the cap-EEG data and twenty to twelve for the ear-EEG data), resampling and filtering, epoch duration selection and base-lining. ICA was used to remove mainly the eye blink components from the data before choosing the best re-referencing option for both recording systems. The ear-EEG data provided the best results using a contralateral reference for each ear channel, this increased the distance between the reference and the channels. For the cap-EEG data, the nose reference was kept as it was the furthest from strong noise contamination from the concurrent recording systems which affected several cap-EEG channels around the ears at different locations for different participants and in intermittent fashion. Linked mastoid reference was rejected because of this unpredictable noise, most likely originating from the ear-EEG equipment. It might also have had an impact by propagation on the use of a common average reference which strongly impacted individual subject differences and PSD distributions at individual channel locations, making it harder to keep a consistent channel selection over all participants and tasks. The nose-reference although not well suited for source localization (Mahjoory et al., 2017) produced more consistent results for inter-subject comparisons of specific electrode locations. Topographical representation of PSD can be biased by a nose reference because of the proximity with ocular artefact location (Trujillo et al., 2005), therefore conclusions regarding topographical data from this study were tested against common average reference topographical representations and held up.

Stronger classification algorithms might be necessary to identify the important features shared between individuals and normalization of the spectral data will most likely be a determining factor in improving this process. In order for the algorithms to be able to generalize across individuals, selecting which frequency ranges are used as features could be very relevant as well as better standardization of the features across subjects through the use of frequency band ratios for example. On model performance comparisons, we have learned from this study that higher spectral resolution tends to produce better classification results at the individual subject level, as does a higher cut-off frequency. However, increasing spatial resolution increases the dimensionality of each sample increasing the risk of over-fitting. This explains why even though the 0.5 Hz resolution models performed better than the 1Hz resolution, the later models were used for visualization and for single trial classification since the final number of features was more appropriate considering our sample size. The choice of a 100 Hz cut-off frequency was influenced by the feature resolution as this model performed significantly better with this cut-off frequency for both tasks. It might be interesting to do future research on maximum useful frequency cut-off as ear-EEG channel features in particular seem like they would be significant past this 100Hz limit. Lastly, the study of temporal features showed that even though they were capable of predicting with above chance-level accuracy the levels of cognitive demand for both recording equipment and channel subsets, they performed worse than spectral ones. Results of this study have shown that while ear-EEG spectral features performed on par with cap-EEG spectral features, ear-EEG temporal data is much noisier than cap-EEG's temporal data as evidenced by the grand average ERP waveforms (Figure 7) and the temporal heatmaps (Figure 8). Dry in-ear EEG channels did not produce a grand average P300 response but were sometimes better than selected cap-EEG channels at differentiating PSD between conditions. Hence ear-EEG might be better suited for applications that rely on spectral data rather than temporal responses, such as focus monitoring. It is worth noting that mixing temporal with spectral features did improve classification results compared to spectral features alone. Although given the abnormally high performance of this type of fusion model (near 100 %), and the high dimensionality of these models, over-fitting is suspected to have happened, despite feature reduction. This will be decisive if these results are to be exploited in a BCI context, it would also make sense to report information transfer rates along with classification accuracies.

Future research should also focus on improving ear-EEG electrode technology. The TIPtrodes^TM^ are very sensitive to movement, which makes them single-use and susceptible to various sources of electromagnetic noise. Around-the-ear electrodes used in this study were wet, innovation in electrode material and treatment could allow their use as dry electrodes. Movement-tolerance of such ear-EEG recording system will surely be a research area of interest going forward. Tighter contact, possibly achieved through custom-fitted earpieces and shielding technologies, could effectively decrease the potential impact of movement artifacts when developing these applications for daily life situations.

## 5. Conclusion

Focus can be assessed using EEG spectral features that were recorded from sites in and around-the-ear using a mobile EEG amplifier. These features are consistent with known characteristics of working memory and cognitive workload. Ear-EEG is a promising candidate for future BCI and brain-monitoring applications, which could help with our day-to-day activities, such as workplace security and productivity.

## Data availability statement

The dataset was meant for use in this study and not for further studies. Requests to access the datasets should be directed to gcretot@critias.ca.

## Ethics statement

The studies involving human participants were reviewed and approved by Ethics Committee of Oldenburg University. The patients/participants provided their written informed consent to participate in this study. Written informed consent was obtained from the individual(s) for the publication of any potentially identifiable images or data included in this article.

## Author contributions

SD, MD, and JV devised the project and the main conceptual ideas. SD validated the experimental paradigms proposed by MB and GC-R. GC-R carried out the experiment and data processing, performed the analytic calculations, developed the statistical analysis and the classification algorithms, and drafted the manuscript and the figures. MD outlined the theoretical formalism and analytic calculations. MD, SD, and MB verified the article's content and added to the analysis and interpretation of the data and conclusions and together with JV they reviewed the final manuscript. JV and MD were co-supervising the project. All authors contributed to the article and approved the submitted version.
